# Spectral- and Temperature-Dependent Phototaxis of *Bemisia tabaci* and Its Predator *Serangium japonicum*: Implications for Predator-Aware Selective LED-Based Trapping

**DOI:** 10.3390/insects17070661

**Published:** 2026-06-25

**Authors:** Xiang Zhang, Zi-Qi He, Pei-Ping Xu, Bao-Li Qiu, Li-He Zhang

**Affiliations:** Engineering Research Center of Biotechnology for Active Substances, College of Modern Mountain Smart Agriculture, Chongqing Normal University, Ministry of Education, Chongqing 401331, China; 17323713347@163.com (X.Z.); curly_xu@163.com (P.-P.X.); baoliqiu@cqnu.edu.cn (B.-L.Q.)

**Keywords:** *Bemisia tabaci*, *Serangium japonicum*, phototaxis, LED, wavelength, temperature

## Abstract

LED-based trapping may improve whitefly management, but light conditions that attract pests may also affect beneficial predators. This study evaluated how wavelength, irradiance, and temperature influence the phototactic behavior of *Bemisia tabaci* Middle East-Asia Minor 1 (MEAM1) and its predator *Serangium japonicum* (Coleoptera: Coccinellidae). Adults were tested in Y-tube assays across multiple wavelength–irradiance combinations at 25 ± 1 °C, followed by temperature-dependent experiments at 20, 25, 30, and 35 °C under selected settings. *B. tabaci* MEAM1 consistently showed strong positive phototaxis. At irradiances of 100, 200, 400, 600, and 800 µW cm^−2^, positive phototaxis was highest under 480 nm light, whereas at 1000 µW cm^−2^, it was highest under 520 nm light. For *S. japonicum*, negative phototaxis was highest under 440 nm at 200 µW cm^−2^ and under 400 nm at 600, 800, and 1000 µW cm^−2^, and this avoidance weakened as temperature increased. Under a strict predator-safe criterion, 400 nm at 600 µW cm^−2^ was the best coordinated setting at 20–30 °C, whereas 440 nm at 600 µW cm^−2^ was the best strict predator-safe setting at 35 °C. These results indicate that selective LED trapping should be deployed as a temperature-aware strategy rather than as a single fixed setting. The findings provide a basis for improving the compatibility of physical and biological control in *B. tabaci* management.

## Simple Summary

Light-emitting diode (LED) traps may help manage the whitefly *Bemisia tabaci*, but they may also affect beneficial insects that naturally suppress this pest. In this study, we examined whether specific LED settings could efficiently trap the pest while reducing unwanted effects on its predator, the predatory ladybird *Serangium japonicum* (Coleoptera: Coccinellidae), and whether this balance changed with temperature. Using Y-tube assays, we compared the responses of the pest and predator to different wavelengths, irradiances, and temperatures. *B. tabaci* was generally attracted, whereas *S. japonicum* showed its strongest avoidance under 400–440 nm light, and this avoidance weakened as temperature increased. These results indicate that selective LED trapping cannot rely on a single fixed setting. Instead, trap parameters should be adjusted according to both optical conditions and temperature when LEDs are used together with biological control. This may improve pest suppression while reducing unintended effects on beneficial insects.

## 1. Introduction

*Bemisia tabaci* (Gennadius) (Hemiptera: Aleyrodidae) is a globally important pest complex that causes direct feeding damage and transmits numerous plant viruses, leading to serious losses in vegetables, cotton, and protected-cropping systems [1,2]. Its management remains challenging because populations can increase rapidly, virus pressure accumulates over the crop cycle, and repeated insecticide applications can promote resistance and disrupt integrated pest management (IPM) programs [1,2]. Environmentally compatible and more precise alternatives to conventional chemical control are therefore increasingly needed. In this context, physical and biological control can serve as important complements or alternatives. In protected agriculture and greenhouse production, combining these two approaches has become an important strategy for the sustainable management of *B. tabaci* [3].

Among physical control methods, light-based trapping has received increasing attention because visual cues play important roles in host location, orientation, dispersal, and trap entry in many pest insects, including whiteflies [4,5,6,7,8,9]. Light-emitting diode (LED) technology offers several practical advantages over conventional light sources and color-based traps, including narrow spectral bandwidths, stable output, low energy consumption, and precise control of wavelength, irradiance, timing, and emitter arrangement [4,5,6,7,8,9]. These properties make LEDs well suited to programmable pest-management applications. Importantly, insect phototactic responses are often shaped by both spectral quality and stimulus intensity rather than by wavelength alone, so wavelength and irradiance should be considered together when optimizing traps [10,11]. Recent studies have shown that LED-based systems can improve monitoring or trapping performance in greenhouse and protected environments and can help identify wavelengths of practical value for whitefly management [12,13,14]. Even so, LED-based trapping still has limitations in practical use. Under some wavelengths or irradiances, both target pests and natural enemies may show positive phototaxis. This may enhance physical control while reducing the effectiveness of biological control, thereby weakening the overall benefits of integrated green pest management. For this reason, practical LED deployment requires identification of optical settings that are suitable not only for the target pest, but also for its key natural enemies.

This issue is particularly important in whitefly systems. Whiteflies are highly responsive to visual cues, and visual manipulation can affect dispersal, orientation, and trap entry [15,16,17]. At the same time, lighting conditions can alter the movement, searching behavior, establishment, and biological-control performance of key natural enemies of *B. tabaci* [18,19,20]. *Serangium japonicum* (Coleoptera: Coccinellidae), a predatory ladybird, is a specialized and important predator of whiteflies, including the pest species *B. tabaci* and other whitefly species such as *Dialeurodes citri* and *Trialeurodes vaporariorum*. It has high predation capacity and can provide sustained biological control. Previous studies have documented its host-plant relationships, natural-enemy associations, population performance, oviposition preference, and seasonal dynamics in whitefly systems [21,22,23,24,25,26]. A comparative framework that evaluates both the pest and its natural enemy is therefore essential for judging the practical value of LED-based trapping. When LEDs are used against *B. tabaci*, it is important to determine how wavelength and irradiance affect not only the pest, but also its predator *S. japonicum*. In practice, improving pest attraction alone is not sufficient for trap design within IPM, and temperature is another key factor that may affect trapping performance.

Because insects are ectothermic, temperature strongly affects locomotor activity, behavioral thresholds, responsiveness to sensory cues, and the timing of movement [4]. As a result, the selectivity of a given wavelength–irradiance combination may vary across thermal conditions. A setting that appears suitable at one temperature may become less selective at another, either because pest attraction weakens or because the predator becomes less avoidant or more likely to enter the illuminated arm. This issue is especially relevant under greenhouse and field conditions, where insects experience substantial diurnal and seasonal temperature variation. Evaluating phototactic responses without considering temperature may therefore overestimate the general applicability of an apparently optimal LED setting.

Although previous studies have documented LED responses in whiteflies, other pest insects, and several natural enemies, important gaps remain [27,28,29]. First, many studies have focused mainly on the target pest, whereas beneficial insects have received less attention. Second, wavelength and irradiance have often been examined separately, even though they may interact in shaping behavioral outcomes. Third, directly comparable datasets for a pest species and its natural enemy under the same assay framework remain limited, especially across different temperatures. Consequently, there is still insufficient evidence to identify lighting conditions that simultaneously favor pest attraction and predator conservation.

In this study, we used *B. tabaci* Middle East-Asia Minor 1 (MEAM1) and its predator *S. japonicum* as a model pest–natural enemy pair to evaluate adult phototactic responses across a range of LED wavelengths and irradiances and to determine how temperature modifies these responses under selected settings. Specifically, we asked three questions: which wavelength–irradiance combinations maximize attraction of *B. tabaci*, which combinations favor avoidance or reduced attraction in *S. japonicum*, and how temperature reshapes the selective window for coordinating LED trapping with biological control. By analyzing both species within the same Y-tube assay framework and by applying explicit decision rules based on replicate-level responses and model-supported comparisons, we aimed to identify an application-oriented parameter space for selective LED deployment in whitefly IPM.

## 2. Materials and Methods

### 2.1. Host Plants and Insect Colonies

Cotton (*Gossypium* spp.; Malvales: Malvaceae, cv. Lumianyan 37) seedlings were grown in pots containing nutrient soil mixed with loess and were used at the 7–9 leaf stage. All host plants and insect colonies were maintained in an insectary at 26 ± 1 °C, 70–80% relative humidity, and a 14:10 h light:dark photoperiod. *Bemisia tabaci* was collected from the teaching farm of South China Agricultural University, confirmed as MEAM1 by mtCOI sequencing, and maintained on cotton for more than ten generations before the experiments. *Serangium japonicum* was maintained as a laboratory colony on *B. tabaci* nymphs, with developmental stages separated to reduce cannibalism. Adults used in the experiments were collected after mating and sexed.

### 2.2. Spectral and Intensity-Dependent Phototactic Responses of B. tabaci and S. japonicum

**Figure 1:**
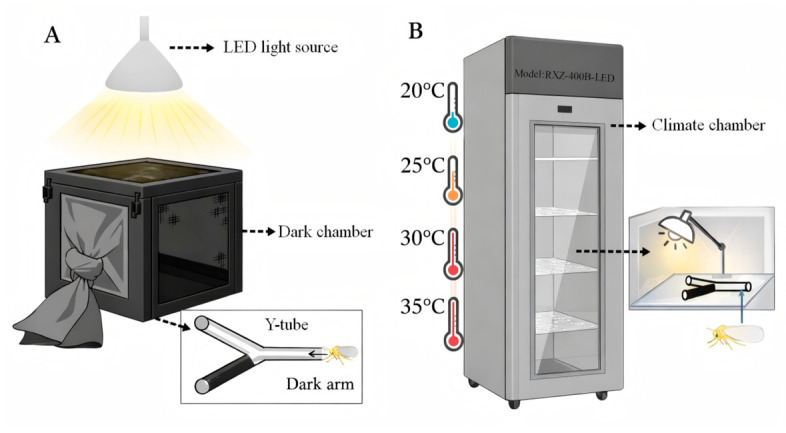
Schematic diagrams of the experimental setups used in the phototaxis assays. (**A**) Overall setup for the wavelength–irradiance assay, with the LED light source positioned above the apparatus to provide overall illumination and the Y-tube used for behavioral testing. (**B**) Temperature-controlled setup used for the phototaxis assay at 20, 25, 30, and 35 °C. Arrows indicate the main components of the experimental apparatus, including the LED light source, dark chamber, Y-tube, dark arm, and climate chamber. The climate chamber model was RXZ-400B-LED. Unless otherwise stated, LED wavelength and irradiance are reported in nm and µW cm^−2^, respectively.

Phototactic behavior was tested in a dark box (90.00 cm × 61.50 cm × 61.50 cm) using a transparent glass Y-tube apparatus supplied by Guangzhou Qianhui Chemical Glass Instrument Co., Ltd. (Guangzhou, China). The apparatus consisted of a base arm, a light arm, and a dark arm. According to the Y-tube apparatus parameters used in the referenced phototaxis assay, each arm was 7.00 cm long, with an inner diameter of 1.80 cm and an outer diameter of 2.20 cm. The Y-tube and LED light source were placed inside the dark box. Except for the experimental LED stimulus, no external light was present. The light arm and dark arm were kept at the same height and had the same visual background; no directional airflow was introduced into either arm, thereby minimizing non-light interference. The experimental setup is shown in Figure 1, with the wavelength–irradiance assay illustrated in Figure 1A.

The light sources were custom-made, round 20 W wavelength-specific LED lamps with conical lamp housings, supplied by Shenzhen Xinhongxian Optoelectronics Technology Co., Ltd. (Shenzhen, China). The nominal wavelengths were those of the custom-made LED units. Different irradiance levels at each wavelength were obtained by adjusting the LED output until the target irradiance was reached. Irradiance was measured using an FZ-A handheld radiometer (Beijing Normal University Optoelectronic Instrument Factory, Beijing, China). For each treatment, the LED was switched on inside the dark box, and the irradiance field was scanned with the radiometer. The measurement point was set at the middle of the light arm of the Y-tube, where the strongest irradiance was detected, and the LED-to-measurement-point distance was 60 cm. The radiometer reading was allowed to stabilize before the LED output was adjusted to the required irradiance and recorded.

Experiments were conducted at 25 ± 2 °C and 70 ± 5% relative humidity between 18:00 and 23:00. Although the assays were performed indoors, the insects were maintained under a 14:10 h light:dark photoperiod and may retain endogenous circadian rhythms. Therefore, this fixed late-day to early-night testing window was used to standardize the circadian background across treatments, to match a period relevant to light-response behavior, and to reduce variation caused by daytime laboratory activity and stray-light disturbance. Fifty pairs of newly emerged *B. tabaci* adults and twenty-five pairs of *S. japonicum* adults were used per replicate. All insects were dark-adapted for 30 min in centrifuge tubes under complete darkness (0 µW cm^−2^) before testing. After adaptation, the containers were connected to the base arm of the Y-tube and all interfaces were sealed. Each treatment lasted 1 h and was replicated in three independent experimental runs rather than as three Y-tubes operated simultaneously. After exposure, the number of insects in the light arm, dark arm, and base arm was recorded. To avoid odor interference, the Y-tube was replaced between treatments, cleaned with distilled water and 75% ethanol, and dried before reuse.

### 2.3. Temperature Effects on Phototactic Responses Under Selected Light Conditions

Based on the wavelength–irradiance screening results and GLM-supported response patterns, selected LED settings were used for the temperature experiments: 400 nm at 600 µW cm^−2^, 440 nm at 600 µW cm^−2^, 480 nm at 400 and 800 µW cm^−2^, 520 nm at 400 and 800 µW cm^−2^, 600 nm at 1000 µW cm^−2^, and 640 nm at 1000 µW cm^−2^. Candidate settings were chosen to represent three biologically relevant groups: short-wavelength treatments with strong predator avoidance, blue-green treatments with strong whitefly attraction, and longer-wavelength comparison treatments that showed relatively high whitefly responses in the screening stage. Selection was made at the treatment-group level rather than from an unqualified single maximum or minimum mean. When numerical means were close or not statistically separated, the corresponding treatments were interpreted as comparable candidates. The temperature-controlled setup is shown in Figure 1B. Experiments were conducted at 20, 25, 30, and 35 °C in a climate chamber with relative humidity maintained at 70 ± 5%. Temperature was continuously monitored, and assays began only after stabilization within 1 °C. All other procedures followed Section 2.2. Each treatment was replicated three times, using 50 pairs of *B. tabaci* adults and 25 pairs of *S. japonicum* adults per replicate. Experiments were conducted between 18:00 and 23:00, using the same fixed daily testing window described above to minimize potential time-of-day effects.

### 2.4. Statistical Analysis

Raw data were analyzed in Python 3.12 using pandas, NumPy, and statsmodels. Data were recorded at the replicate level as counts in the light arm, dark arm, and base arm. In the formulas below, L denotes the number of insects in the light arm, D denotes the number in the dark arm, B denotes the number remaining in the base arm, and N denotes the total number of tested insects. The total number of individuals was calculated as:

N = L + D + B

Positive phototaxis was calculated as L/N × 100, negative phototaxis as D/N × 100, and response rate as (L + D)/N × 100.

For descriptive analysis, replicate-level percentages were summarized as mean ± standard error, with treatment means shown in the heatmaps and replicate-level values provided in Tables S1 and S2. In the wavelength and irradiance screening experiments, binomial generalized linear models with a logit link were fitted separately for *B. tabaci* and *S. japonicum*, with wavelength, irradiance, and their interaction as fixed effects. In the temperature experiments, binomial generalized linear models included temperature, LED setting, and their interaction as fixed effects. Observed total counts were used as the binomial denominator. Significance was evaluated using Wald chi-square tests, with *p* < 0.05 considered significant. For treatment-level interpretation, model-based pairwise contrasts were used with Holm adjustment for multiple comparisons. Results described as higher, lower, or comparable among treatments were interpreted according to the GLM and adjusted multiple-comparison results rather than by absolute mean values alone. Omnibus Wald chi-square results are provided in Table S3.

For treatment selection, candidate whitefly-attractive settings were first identified from treatments with high *B. tabaci* positive phototaxis, low negative phototaxis, and favorable model-supported groupings. Candidate predator-safe settings were identified when *S. japonicum* negative phototaxis exceeded positive phototaxis, and the setting showed relatively high predator avoidance in the model-supported comparisons. Ranking points were then assigned separately for whitefly attraction and predator safety and combined to define coordinated candidate settings. This ranking procedure was used as a decision aid for IPM-oriented parameter selection, not as a replacement for the GLM and multiple-comparison results. Temperature-specific ranking outputs are presented in Tables S4 and S5, candidate coordinated-setting scores in Table S6, and supplementary behavioral metrics and decision rules in Table S7.

## 3. Results

### 3.1. Spectral-Irradiance Responses of Bemisia tabaci

**Figure 2:**
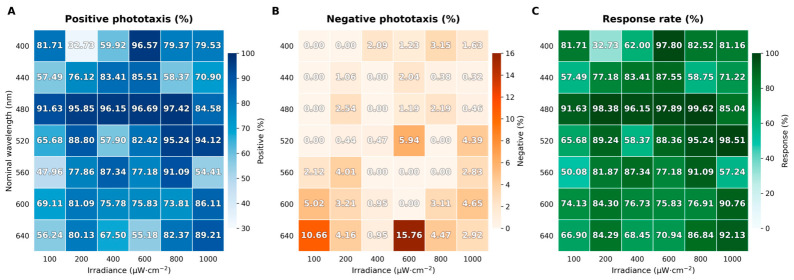
Heatmap summary of phototactic responses of *Bemisia tabaci* MEAM1 adults across nominal LED wavelength (nm) and irradiance (µW cm^−2^) combinations. (**A**) Positive phototaxis (%), (**B**) negative phototaxis (%), and (**C**) response rate (%). Values in cells are treatment means (*n* = 3). Raw data are provided in Table S1.

Across all wavelength–irradiance combinations, *B. tabaci* MEAM1 showed consistently strong positive phototaxis, although the wavelength associated with the strongest attraction varied with irradiance (Figure 2A; Table 1). Binomial GLMs confirmed significant effects of wavelength, irradiance, and their interaction on positive phototaxis (all *p* < 0.001; Table S3). At 100–800 µW cm^−2^, 480 nm belonged to the leading attraction candidates and numerically produced the largest positive phototaxis means, with values exceeding 91% and reaching 97.42% at 800 µW cm^−2^. At 600 µW cm^−2^, 400 and 480 nm produced comparable responses, both exceeding 96%, with only a marginal numerical difference of 0.12 percentage points. At 1000 µW cm^−2^, the leading numerical value shifted to 520 nm (94.12%) (Figure 2A; Table 1). Negative phototaxis remained low across all treatments (Figure 2B). GLM results indicated a significant effect of wavelength (χ^2^ = 15.6229, *p* = 0.0159), whereas irradiance (*p* = 0.8538) and the interaction term (*p* = 0.1205) were not significant (Table S3).

Response rates were generally high (Figure 2C), indicating that differences among treatments primarily reflected directional preference rather than differences in activity. Replicate-level screening responses of *B. tabaci* MEAM1 are provided in Figure S1.

### 3.2. Spectral-Irradiance Responses of Serangium japonicum

**Figure 3:**
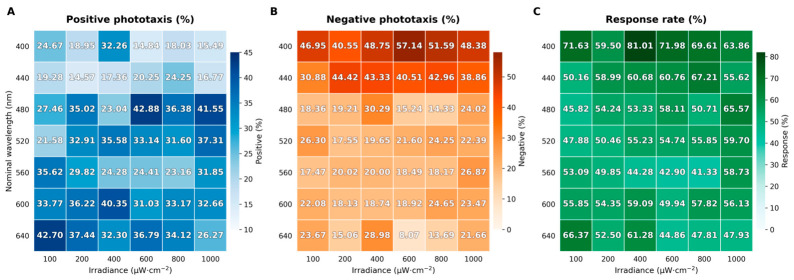
Heatmap summary of phototactic responses of *Serangium japonicum* adults across nominal LED wavelength (nm) and irradiance (µW cm^−2^) combinations. (**A**) Positive phototaxis (%), (**B**) negative phototaxis (%), and (**C**) response rate (%). Values in cells are treatment means (*n* = 3). Raw data are provided in Table S1.

Across the screening matrix, *Serangium japonicum* showed moderate positive phototaxis that varied with both wavelength and irradiance (Figure 3A). Binomial GLMs indicated that predator positive phototaxis was significantly affected by wavelength (*p* < 0.001), irradiance (*p* = 0.0166), and their interaction (*p* < 0.001; Table S3). Model-supported response patterns showed generally stronger positive phototaxis under 480–640 nm than under 400–440 nm, with relatively high numerical values at 480 nm and 640 nm under selected irradiance levels (Figure 3A).

In contrast, negative phototaxis was concentrated at 400 and 440 nm (Figure 3B). At 400 nm, negative phototaxis increased with irradiance and reached 57.14 ± 7.14% at 600 µW cm^−2^. At 440 nm, negative phototaxis peaked numerically at 44.42 ± 6.02% at 200 µW cm^−2^ and remained comparatively high at 400–800 µW cm^−2^. Predator negative phototaxis was significantly affected by wavelength (*p* < 0.001) and the wavelength × irradiance interaction (*p* = 0.0141), whereas irradiance alone was not significant (*p* = 0.2822; Table S3). Together, these patterns identified 400–440 nm as the principal predator-avoidance band in the screening stage.

Response rates remained moderate across treatments (Figure 3C). Under 400–440 nm, a considerable proportion of adults responded but were predominantly distributed in the dark arm. Under several longer-wavelength conditions, the difference between positive and negative phototaxis was reduced or reversed (Figure 3A–C). Replicate-level screening responses of *S. japonicum* are provided in Figure S2.

### 3.3. Temperature Effects on Bemisia tabaci Under Selected Light Conditions

**Figure 4:**
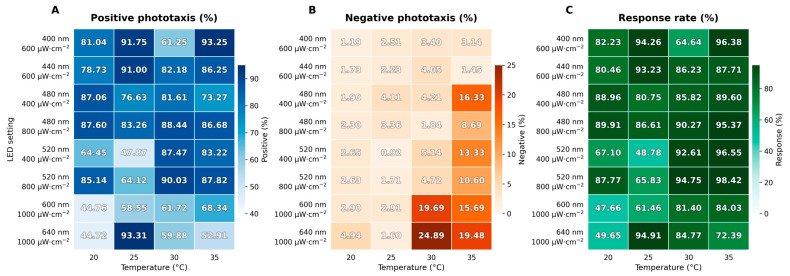
Heatmap summary of temperature-dependent responses of *Bemisia tabaci* MEAM1 adults under selected LED settings (wavelength, nm; irradiance, µW cm^−2^). (**A**) Positive phototaxis (%), (**B**) negative phototaxis (%), and (**C**) response rate (%). Values in cells are treatment means (*n* = 3). Raw data are provided in Table S2.

Temperature altered the ranking of whitefly-attractive settings among the selected LED combinations (Figure 4A; Table 2). Positive phototaxis remained higher than negative phototaxis across all treatments, although the leading attraction candidates varied with temperature. Binomial GLMs showed that whitefly positive phototaxis was significantly affected by temperature, setting, and their interaction (all *p* < 0.001; Table S3).

At 20 °C, 480 nm at 800 µW cm^−2^ (87.60%), 480 nm at 400 µW cm^−2^ (87.06%), and 520 nm at 800 µW cm^−2^ (85.14%) formed the leading attraction candidates. At 25 °C, 640 nm at 1000 µW cm^−2^ ranked first numerically (93.31%), whereas 400 nm at 600 µW cm^−2^ (91.75%) and 440 nm at 600 µW cm^−2^ (91.00%) also showed high positive phototaxis. At 30 °C, 520 nm at 800 µW cm^−2^, 480 nm at 800 µW cm^−2^, and 520 nm at 400 µW cm^−2^ were the leading numerical candidates. At 35 °C, 400 nm at 600 µW cm^−2^ showed the largest numerical value (93.25%), followed by 520 nm at 800 µW cm^−2^ (87.82%) and 480 nm at 800 µW cm^−2^ (86.68%) (Figure 4A). This temperature-dependent shift may reflect changes in locomotor activity and visual response thresholds; as temperature increases, insect activity and sensory responsiveness may broaden or alter the effective attractive range of LED stimuli.

Negative phototaxis remained low across most temperature–setting combinations (Figure 4B). GLM results indicated that whitefly negative phototaxis was driven mainly by the temperature × setting interaction (*p* < 0.001), whereas the main effects of temperature (*p* = 0.4306) and setting (*p* = 0.3250) were not significant (Table S3). Response rate also varied significantly with temperature, setting, and their interaction (all *p* < 0.001; Figure 4C; Table S3). Replicate-level temperature-stage responses of *B. tabaci* MEAM1 are provided in Figure S3.

### 3.4. Temperature Effects on Serangium japonicum Under Selected Light Conditions

**Figure 5:**
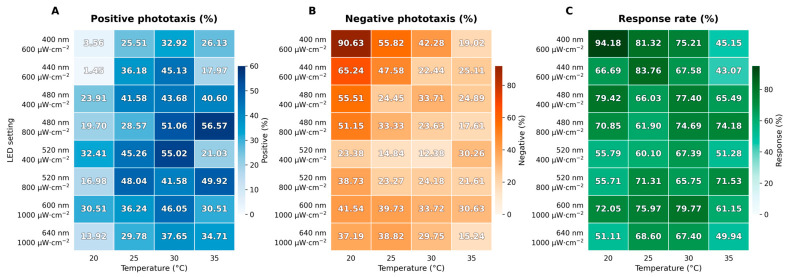
Heatmap summary of temperature-dependent responses of *Serangium japonicum* adults under selected LED settings (wavelength, nm; irradiance, µW cm^−2^). (**A**) Positive phototaxis (%), (**B**) negative phototaxis (%), and (**C**) response rate (%). Values in cells are treatment means (*n* = 3). Raw data are provided in Table S2.

Temperature altered predator responses across the selected nominal LED settings (Figure 5A–C). Binomial GLMs showed that predator positive phototaxis, predator negative phototaxis, and predator response rate were all significantly affected by temperature, setting, and their interaction (all *p* < 0.001; Table S3).

Predator positive phototaxis varied across temperature–setting combinations and generally increased with temperature in several settings, reducing the difference between positive and negative phototaxis (Figure 5A).

By contrast, negative phototaxis was strongest under the near-UV/violet settings at lower temperatures (Figure 5B), and the corresponding temperature-specific predator-safe candidates are summarized in Table 2. At 20 °C, 400 nm at 600 µW cm^−2^ showed the largest numerical negative phototaxis (90.63%), followed by 440 nm at 600 µW cm^−2^ (65.24%). At 25 °C, 400 nm at 600 µW cm^−2^ again ranked first numerically for avoidance, with 440 nm at 600 µW cm^−2^ ranking second. At 30 °C, 400 nm at 600 µW cm^−2^ remained the leading numerical avoidance candidate, although mean negative phototaxis decreased to 42.28%. At 35 °C, 520 nm at 400 µW cm^−2^ showed the largest numerical value (30.26%), followed by 440 nm at 600 µW cm^−2^ (25.11%). For comparison, 600 nm at 1000 µW cm^−2^ showed a similar negative phototaxis (30.63%) but also a comparable level of positive phototaxis (30.51%), which limited its value as a predator-safe setting (Figure 5B). Response rates remained moderate across the selected settings, and replicate-level temperature-stage responses of *S. japonicum* are provided in Figure S4.

## 4. Discussion

The present study shows that *B. tabaci* MEAM1 and its predator *S. japonicum* differ markedly in their phototactic responses to LED conditions, and that temperature further modifies these differences. *B. tabaci* generally showed stronger positive phototaxis to blue-green wavelengths, whereas *S. japonicum* showed avoidance or only weak positive phototaxis within a narrower portion of the spectrum. Importantly, the setting that maximized whitefly attraction was not always the setting that best protected the predator. These findings indicate that LED trapping should be integrated with biological control in a temperature-aware manner rather than applied as a fixed, one-setting strategy.

### 4.1. Spectral Response Patterns and Their Biological Interpretation

The strong attraction of *B. tabaci* to selected blue-green wavelengths is consistent with previous LED-based studies reporting wavelength-dependent attraction in whitefly species [30,31,32] and with the visual ecology of many herbivorous insects [33]. Insect compound eyes contain photoreceptors with different spectral sensitivities, and visually guided host-location behavior is often tuned to wavelengths associated with host plants and the surrounding vegetation background. In hemipteran insects, attraction to green or green-related wavelengths is often interpreted as a response to host-reflected signals, whereas shorter wavelengths may contribute differently depending on visual context and stimulus intensity [33]. In the present study, the high attractiveness of 480–520 nm to *B. tabaci* fits this general framework and supports the view that host-oriented visual behavior is mediated by wavelength-specific sensitivity rather than by nonspecific attraction to light alone.

By contrast, *S. japonicum* showed a different response pattern, with the clearest avoidance concentrated in the near-UV and violet range, especially around 400–440 nm. This suggests that the predator does not simply follow the same visual cues as the pest, but responds to the optical environment in a species-specific way. One possible explanation is that short-wavelength light has a different ecological meaning for predatory coccinellids than for whiteflies, either because of differences in receptor sensitivity, photoreceptor composition, opsin expression, or the behavioral contexts in which short-wavelength cues are used. Although the present study did not directly examine sensory physiology, the behavioral pattern is consistent with the view that the pest and predator occupy different visual niches. This distinction is important for selective trapping because it creates the possibility that some wavelengths may remain attractive to the pest while discouraging predator entry.

These findings are broadly consistent with previous studies showing that whitefly responses to LEDs are wavelength-dependent and that beneficial insects do not respond uniformly across the same spectral range [34,35,36]. At the same time, our results extend this body of work by comparing a pest and its natural enemy within the same assay system. This comparison matters because the practical value of a light source depends not only on how strongly it attracts the target pest, but also on how it affects beneficial insects.

### 4.2. Irradiance Modulates Wavelength-Dependent Responses

Another important finding was that wavelength alone did not fully explain the behavioral response. In *B. tabaci*, the ranking of attractive treatments changed with irradiance, indicating that the effect of a given wavelength depended on light intensity. Although 480 nm performed well across much of the screening range, its advantage was not identical at all irradiance levels, and the best treatment changed at the highest irradiance. This means that an attractive wavelength cannot be evaluated independently of stimulus strength.

This pattern is biologically plausible because irradiance can influence receptor stimulation and response thresholds. When intensity changes, the same wavelength may not be perceived in the same way, and the behavioral outcome may also change. Similar intensity-dependent visual responses have been reported in whiteflies and other insects [6,7,33,36]. Therefore, trap optimization should consider wavelength and irradiance together.

This point also matters in practice. If trap design focuses only on wavelength and ignores irradiance, the selected LED setting may not perform consistently when trap structure or operating conditions change. Our results show that irradiance is not simply a secondary adjustment; it can alter the relative performance of candidate wavelengths.

### 4.3. Temperature Reshapes Both Attraction and Selectivity

Temperature was a key factor affecting the responses observed in this study. Because insects are ectothermic, temperature affects their activity and responsiveness to visual stimuli. Our results showed that both *B. tabaci* and *S. japonicum* changed their responses across temperatures, so the usefulness of a given LED setting was not constant.

In *S. japonicum*, avoidance under predator-safe settings weakened as temperature increased. This means that a setting that works well at a lower temperature may become less selective at a higher temperature. In *B. tabaci*, temperature also changed the relative performance of candidate settings. Therefore, the effects of light conditions should be interpreted together with temperature.

This finding is ecologically important. In greenhouse and field systems, temperature changes across the day and season, and trap performance may also be affected by background light, crop structure, airflow, and other environmental conditions [37,38,39,40,41]. Results obtained at a single laboratory temperature may therefore overestimate how stable an LED setting will be in practice. Our findings suggest that temperature should be taken into account when selecting trap parameters.

### 4.4. Implications for Coordinated Pest Attraction and Predator Conservation

From an IPM perspective, the most important finding was that the LED setting with the strongest attraction to *B. tabaci* was not always the setting that best reduced the risk to *S. japonicum*. Therefore, LED-based trapping should not be optimized only for pest attraction. Instead, candidate settings should be evaluated according to both whitefly attraction and predator avoidance.

At 20–25 °C, 400 nm at 600 μW cm^−2^ provided the clearest coordinated performance because it maintained attraction to *B. tabaci* while inducing strong avoidance in *S. japonicum*. At 30 °C, this setting still met the predator-safe criterion, but the margin between pest attraction and predator protection became smaller. At 35 °C, the coordinated-use window shifted, and 440 nm at 600 μW cm^−2^ appeared more suitable under the strict predator-safe rule.

These results suggest that LED deployment in greenhouse systems should be temperature-aware and predator-conscious. When biological control is used together with LED trapping, priority should be given to settings that maintain pest attraction without increasing predator entry into the illuminated arm. Field or greenhouse validation is still needed before these candidate settings can be used as operational recommendations.

### 4.5. Limitations and Future Directions

Several limitations should be considered when interpreting these findings. First, the experiments were conducted under controlled laboratory conditions using a Y-tube assay. This design allowed standardized comparisons among LED treatments, but it cannot fully represent greenhouse or field environments, where background light, plant architecture, airflow, and competing sensory cues may modify insect behavior. Therefore, the identified LED settings should be regarded as candidate parameters for further validation rather than as final deployment recommendations.

Second, the study focused on one predator species, *S. japonicum*. Although previous studies have described its biological characteristics, host associations, and role in whitefly biological control [42,43], other predators and parasitoids may differ in spectral sensitivity and temperature-dependent responses. The predator-aware settings identified here may therefore not apply equally to all natural-enemy communities.

Third, this study did not examine sex-specific responses, circadian variation, long-term trap catches, or multi-species interactions under crop-production conditions. Future studies should test the candidate LED settings in greenhouse or field trials and evaluate their effects on both pest suppression and natural-enemy conservation, as field-based trap validation remains essential before operational deployment [44].

Overall, this study provides experimental evidence that wavelength, irradiance, and temperature jointly influence the balance between whitefly attraction and predator safety. These results may help guide future optimization of LED trapping in whitefly IPM.

## 5. Conclusions

**Figure 6:**
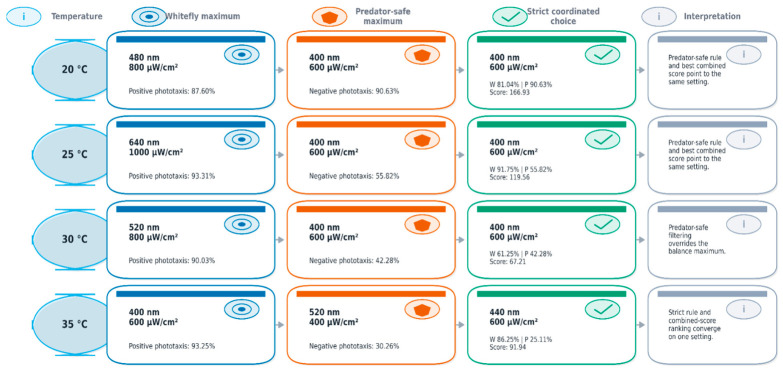
Temperature-specific decision pathway for predator-aware LED deployment across selected LED settings. For each temperature, the diagram compares the leading whitefly-attraction setting, the leading predator-safe setting, and the strict coordinated setting. Percentage values shown in the diagram are treatment means derived from the temperature-stage dataset. LED wavelengths and irradiances are reported in nm and µW cm^−2^, respectively.

Under a common Y-tube assay framework, *B. tabaci* MEAM1 and *S. japonicum* showed clearly different phototactic responses to LED settings, and these differences depended on both irradiance and temperature. The most practical predator-aware selectivity window was observed at 20–25 °C and centered on 400 nm at 600 µW cm^−2^, whereas the coordinated-use margin weakened at 30 °C and shifted toward 440 nm at 600 µW cm^−2^ at 35 °C. Selective LED trapping should therefore be deployed as a temperature-aware strategy that balances whitefly attraction with predator conservation rather than as a fixed protocol. Overall, this study clarifies the joint roles of wavelength, irradiance, and temperature in shaping selective phototactic responses in a pest–natural enemy system and provides a basis for optimizing LED deployment in more precise and biologically compatible whitefly management. The temperature-specific decision pathway is summarized in Figure 6.

## Figures and Tables

**Table 1:** Peak and second-ranked wavelengths for positive phototaxis in *Bemisia tabaci* MEAM1 at each irradiance level in the wavelength screening assay. Values are mean ± SE percentages based on the realized total counted per replicate, *n* = 3; replicate-specific denominators are provided in Table S1.

Irradiance	Peak Wavelength	Peak Positive Phototaxis	Peak Negative Phototaxis	Second-Ranked Wavelength	Second-Ranked Positive Phototaxis	Second-Ranked Negative Phototaxis	Difference
(µW cm^−2^)	(nm)	(%, Mean ± SE)	(%, Mean)	(nm)	(%, Mean ± SE)	(%, Mean)	(Percentage Points)
100	480	91.63 ± 0.68	0.00	400	81.71 ± 2.93	0.00	9.92
200	480	95.85 ± 0.57	2.54	520	88.80 ± 3.92	0.44	7.04
400	480	96.15 ± 2.52	0.00	560	87.34 ± 1.40	0.00	8.81
600	480	96.69 ± 1.52	1.19	400	96.57 ± 0.31	1.23	0.12
800	480	97.42 ± 1.05	2.19	520	95.24 ± 2.81	0.00	2.18
1000	520	94.12 ± 0.92	4.39	640	89.21 ± 0.79	2.92	4.91

**Table 2:** Temperature-specific leading candidate settings for whitefly attraction and predator avoidance under selected LED conditions.

Temperature (°C)	Leading Candidate Setting for Whitefly Attraction	Whitefly Positive Phototaxis (%, Mean)	Leading Candidate Setting for Predator Avoidance	Predator Negative Phototaxis (%, Mean)
20	480 nm at 800 µW cm^−2^	87.60	400 nm at 600 µW cm^−2^	90.63
25	640 nm at 1000 µW cm^−2^	93.31	400 nm at 600 µW cm^−2^	55.82
30	520 nm at 800 µW cm^−2^	90.03	400 nm at 600 µW cm^−2^	42.28
35	400 nm at 600 µW cm^−2^	93.25	520 nm at 400 µW cm^−2^	30.26

## Data Availability

The data that support the findings of this study are available in the Supplementary Materials.

## Supplementary Materials

The following supporting information can be downloaded at: https://www.mdpi.com/article/10.3390/insects17070661/s1; Figure S1: replicate-level screening responses of *Bemisia tabaci* MEAM1 across nominal wavelength–irradiance combinations; Figure S2: replicate-level screening responses of *Serangium japonicum* across nominal wavelength–irradiance combinations; Figure S3: replicate-level temperature-stage responses of *Bemisia tabaci* MEAM1 under selected nominal LED settings; Figure S4: replicate-level temperature-stage responses of *Serangium japonicum* under selected nominal LED settings; Table S1: replicate-level screening counts and derived phototaxis metrics for both species; Table S2: replicate-level temperature-assay counts and derived phototaxis metrics for both species; Table S3: omnibus Wald chi-square tests from the raw-count binomial GLM reanalysis; Table S4: temperature-specific whitefly summary table used for ranking and decision analysis; Table S5: temperature-specific predator summary table used for ranking and decision analysis; Table S6: all candidate coordinated-setting scores before strict decision filtering; Table S7: definitions of supplementary behavioral metrics and detailed decision rules used for ranking candidate LED settings. Source files are packaged in the supplementary figure and table directories together with the scripts used to generate the master tables, GLM outputs, and final figures.

## Abbreviations

The following abbreviations are used in this manuscript:

GLM	Generalized linear model
IPM	Integrated pest management
LED	Light-emitting diode
MEAM1	Middle East-Asia Minor 1
RH	Relative humidity
SD	Standard deviation
UV	Ultraviolet
