# Supplementary material for: Spectral- and Temperature-Dependent Phototaxis of Bemisia tabaci and Its Predator Serangium japonicum: Implications for Predator-Aware Selective LED-Based Trapping
Source: Insects. 2026 Jun 25;17(7):661. doi: 10.3390/insects17070661 (PMC13410480; doi:10.3390/insects17070661)
Supplement: Supplementary file 1 [file insects-17-00661-s001.zip › insects-4361364-supplementary.pdf]

Supplementary Materials

For: Spectral- and Temperature-Dependent Phototaxis of Bemisia tabaci and Its Predator Serangium japonicum: Implications for Predator-Aware Selective LED-Based Trapping

Contents

Supplementary Figures: Figure S1-Figure S4

Supplementary Tables: Table S1-Table S7

Supplementary Figures

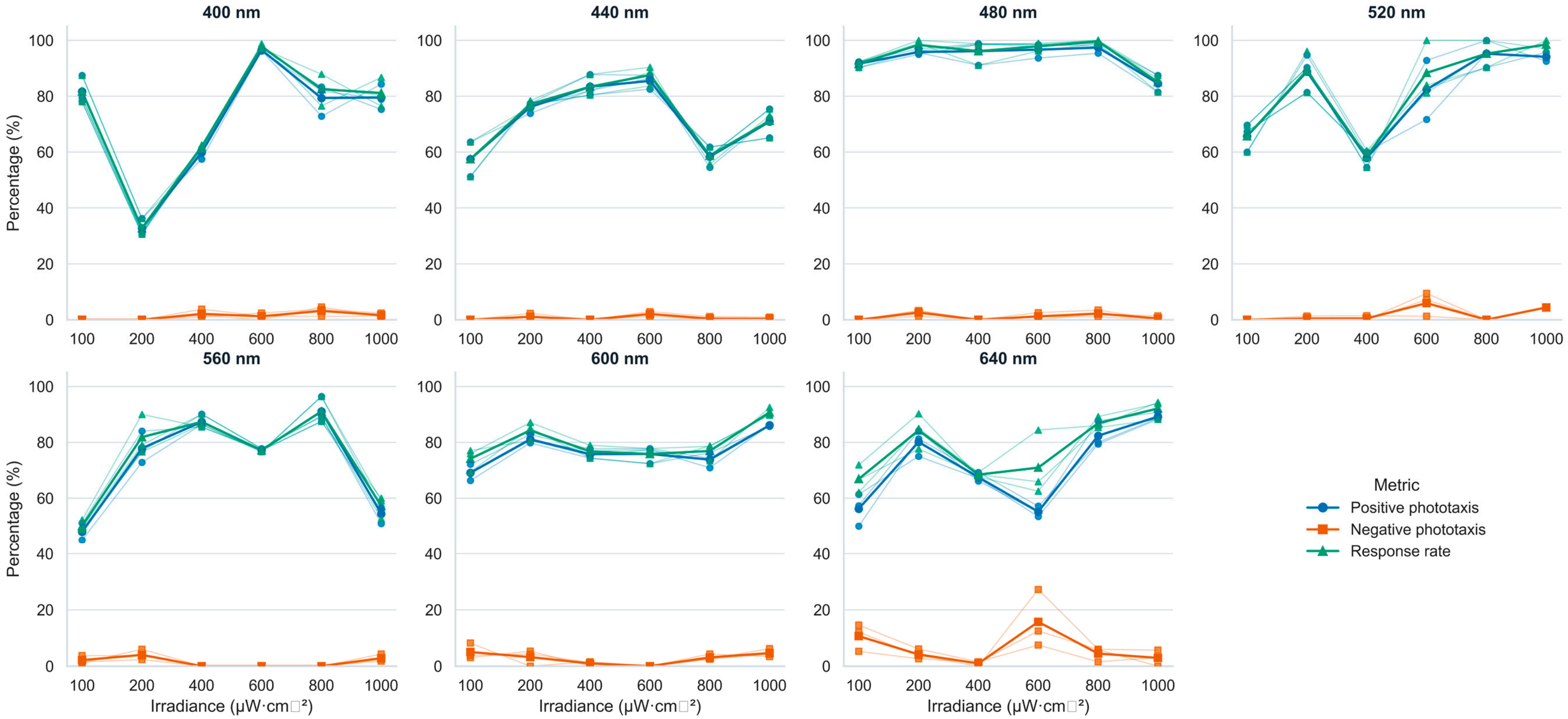

Figure S1. Replicate-level screening responses of Bemisia tabaci MEAM1 across nominal wavelength-irradiance combinations.

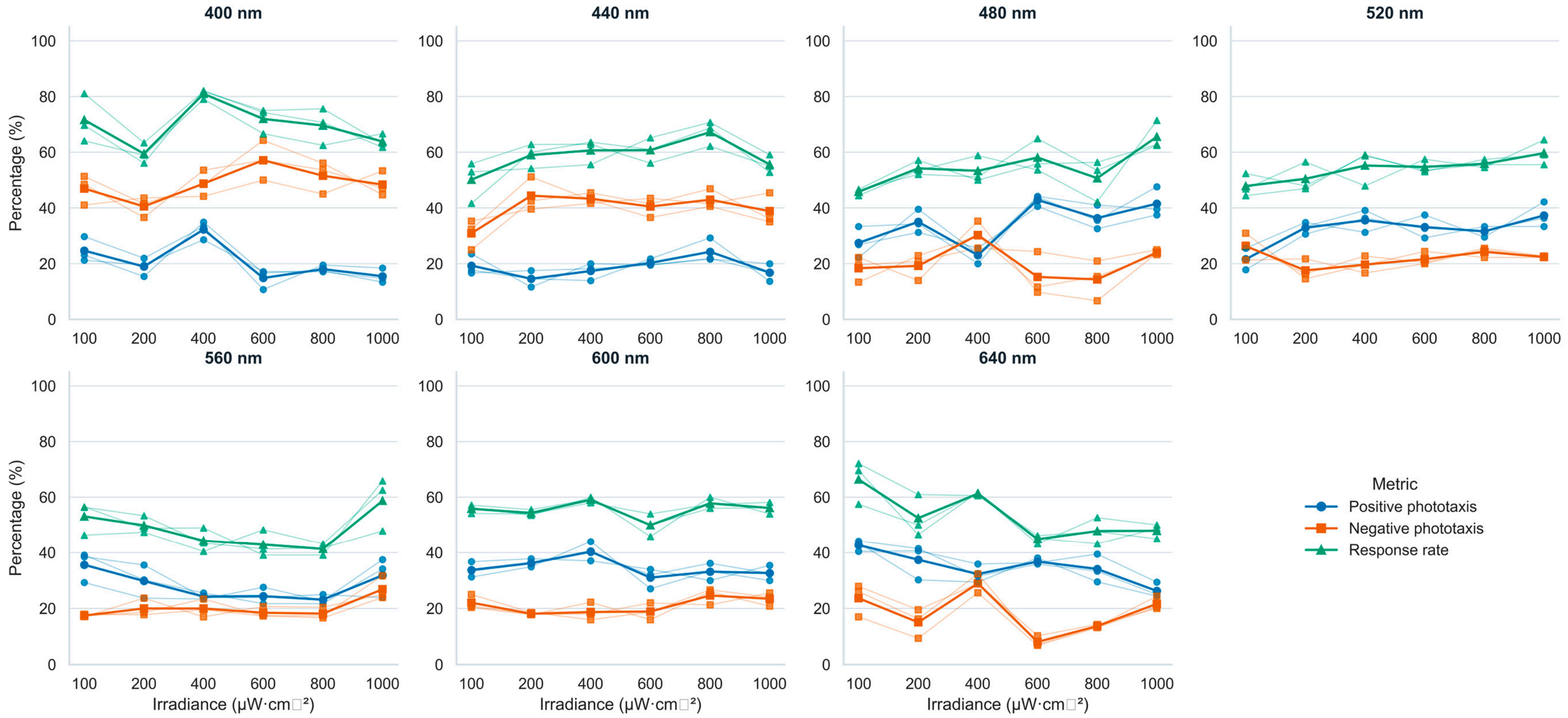

Figure S2. Replicate-level screening responses of Serangium japonicum across nominal wavelength-irradiance combinations.

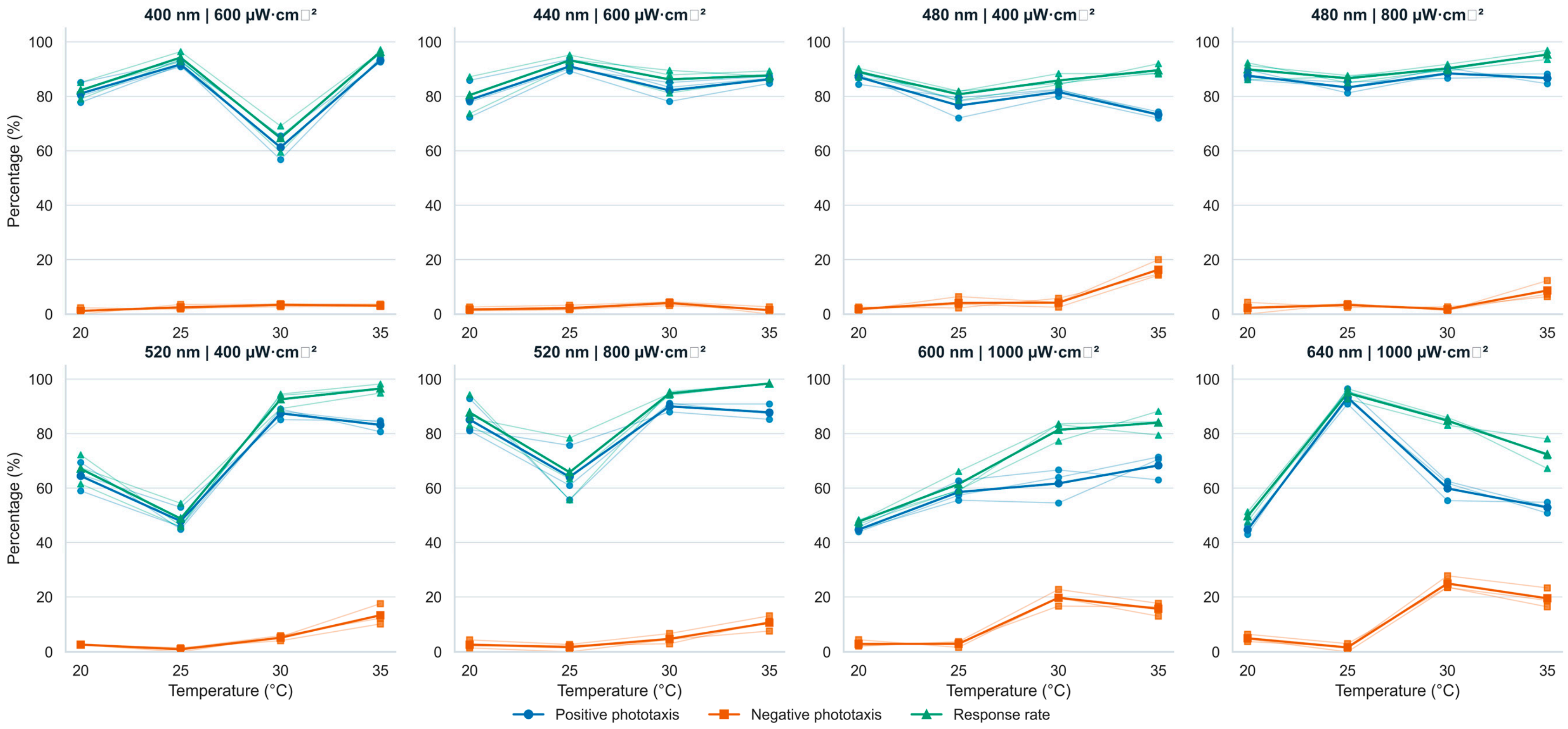

Figure S3. Replicate-level temperature-stage responses of Bemisia tabaci MEAM1 under selected nominal LED settings.

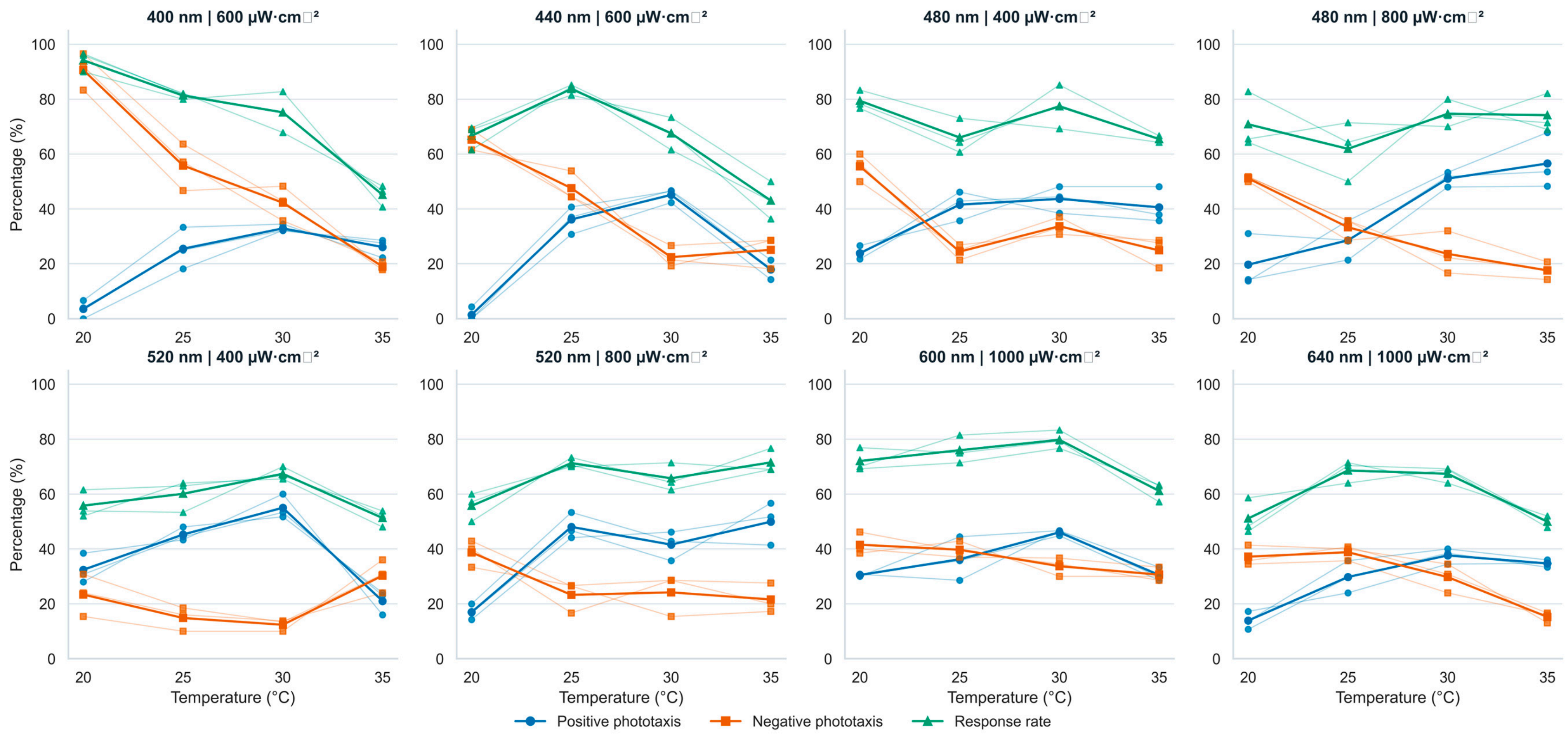

Figure S4. Replicate-level temperature-stage responses of Serangium japonicum under selected nominal LED settings.

Supplementary Tables

Table S1. Replicate-level screening counts and derived phototaxis metrics for both species.

Assay type	Species	Wavelength (nm)	Irradiance (μW cm^{-2})	Replicate id	Actual total (n)	Light arm (n)	Dark arm (n)	Base arm (n)	Positive phototaxis (%)	Negative phototaxis (%)	Response rate (%)	Light preference among responders (%)	Choice index among responders
Screening	Whitefly	400.00	100.00	1.00	88.00	70.00	0.00	18.00	79.55	0.00	79.55	100.00	1.00
Screening	Whitefly	400.00	100.00	2.00	88.00	77.00	0.00	11.00	87.50	0.00	87.50	100.00	1.00
Screening	Whitefly	400.00	100.00	3.00	73.00	57.00	0.00	16.00	78.08	0.00	78.08	100.00	1.00
Screening	Whitefly	400.00	200.00	1.00	67.00	21.00	0.00	46.00	31.34	0.00	31.34	100.00	1.00
Screening	Whitefly	400.00	200.00	2.00	69.00	25.00	0.00	44.00	36.23	0.00	36.23	100.00	1.00
Screening	Whitefly	400.00	200.00	3.00	49.00	15.00	0.00	34.00	30.61	0.00	30.61	100.00	1.00
Screening	Whitefly	400.00	400.00	1.00	64.00	39.00	1.00	24.00	60.94	1.56	62.50	97.50	0.95
Screening	Whitefly	400.00	400.00	2.00	80.00	46.00	3.00	31.00	57.50	3.75	61.25	93.88	0.88
Screening	Whitefly	400.00	400.00	3.00	106.00	65.00	1.00	40.00	61.32	0.94	62.26	98.48	0.97
Screening	Whitefly	400.00	600.00	1.00	71.00	69.00	0.00	2.00	97.18	0.00	97.18	100.00	1.00
Screening	Whitefly	400.00	600.00	2.00	78.00	75.00	1.00	2.00	96.15	1.28	97.44	98.68	0.97
Screening	Whitefly	400.00	600.00	3.00	83.00	80.00	2.00	1.00	96.39	2.41	98.80	97.56	0.95
Screening	Whitefly	400.00	800.00	1.00	66.00	55.00	3.00	8.00	83.33	4.55	87.88	94.83	0.90
Screening	Whitefly	400.00	800.00	2.00	83.00	68.00	1.00	14.00	81.93	1.20	83.13	98.55	0.97
Screening	Whitefly	400.00	800.00	3.00	81.00	59.00	3.00	19.00	72.84	3.70	76.54	95.16	0.90
Screening	Whitefly	400.00	1000.00	1.00	85.00	64.00	1.00	20.00	75.29	1.18	76.47	98.46	0.97

Assay type	Species	Wavelength (nm)	Irradiance (μW cm⁻²)	Replicate id	Actual total (n)	Light arm (n)	Dark arm (n)	Base arm (n)	Positive phototaxis (%)	Negative phototaxis (%)	Response rate (%)	Light preference among responders (%)	Choice index among responders
Screening	Whitefly	400.00	1000.00	2.00	76.00	60.00	1.00	15.00	78.95	1.32	80.26	98.36	0.97
Screening	Whitefly	400.00	1000.00	3.00	83.00	70.00	2.00	11.00	84.34	2.41	86.75	97.22	0.94
Screening	Whitefly	440.00	100.00	1.00	99.00	57.00	0.00	42.00	57.58	0.00	57.58	100.00	1.00
Screening	Whitefly	440.00	100.00	2.00	80.00	41.00	0.00	39.00	51.25	0.00	51.25	100.00	1.00
Screening	Whitefly	440.00	100.00	3.00	66.00	42.00	0.00	24.00	63.64	0.00	63.64	100.00	1.00
Screening	Whitefly	440.00	200.00	1.00	87.00	67.00	0.00	20.00	77.01	0.00	77.01	100.00	1.00
Screening	Whitefly	440.00	200.00	2.00	111.00	86.00	1.00	24.00	77.48	0.90	78.38	98.85	0.98
Screening	Whitefly	440.00	200.00	3.00	88.00	65.00	2.00	21.00	73.86	2.27	76.14	97.01	0.94
Screening	Whitefly	440.00	400.00	1.00	97.00	78.00	0.00	19.00	80.41	0.00	80.41	100.00	1.00
Screening	Whitefly	440.00	400.00	2.00	90.00	79.00	0.00	11.00	87.78	0.00	87.78	100.00	1.00
Screening	Whitefly	440.00	400.00	3.00	78.00	64.00	0.00	14.00	82.05	0.00	82.05	100.00	1.00
Screening	Whitefly	440.00	600.00	1.00	86.00	71.00	1.00	14.00	82.56	1.16	83.72	98.61	0.97
Screening	Whitefly	440.00	600.00	2.00	104.00	91.00	3.00	10.00	87.50	2.88	90.38	96.81	0.94
Screening	Whitefly	440.00	600.00	3.00	96.00	83.00	2.00	11.00	86.46	2.08	88.54	97.65	0.95
Screening	Whitefly	440.00	800.00	1.00	89.00	55.00	0.00	34.00	61.80	0.00	61.80	100.00	1.00
Screening	Whitefly	440.00	800.00	2.00	88.00	48.00	1.00	39.00	54.55	1.14	55.68	97.96	0.96
Screening	Whitefly	440.00	800.00	3.00	97.00	57.00	0.00	40.00	58.76	0.00	58.76	100.00	1.00
Screening	Whitefly	440.00	1000.00	1.00	86.00	56.00	0.00	30.00	65.12	0.00	65.12	100.00	1.00
Screening	Whitefly	440.00	1000.00	2.00	104.00	75.00	1.00	28.00	72.12	0.96	73.08	98.68	0.97

Assay type	Species	Wavelength (nm)	Irradiance (μW cm^{-2})	Replicate id	Actual total (n)	Light arm (n)	Dark arm (n)	Base arm (n)	Positive phototaxis (%)	Negative phototaxis (%)	Response rate (%)	Light preference among responders (%)	Choice index among responders
Screening	Whitefly	440.00	1000.00	3.00	110.00	83.00	0.00	27.00	75.45	0.00	75.45	100.00	1.00
Screening	Whitefly	480.00	100.00	1.00	72.00	65.00	0.00	7.00	90.28	0.00	90.28	100.00	1.00
Screening	Whitefly	480.00	100.00	2.00	65.00	60.00	0.00	5.00	92.31	0.00	92.31	100.00	1.00
Screening	Whitefly	480.00	100.00	3.00	65.00	60.00	0.00	5.00	92.31	0.00	92.31	100.00	1.00
Screening	Whitefly	480.00	200.00	1.00	80.00	76.00	1.00	3.00	95.00	1.25	96.25	98.70	0.97
Screening	Whitefly	480.00	200.00	2.00	91.00	87.00	3.00	1.00	95.60	3.30	98.90	96.67	0.93
Screening	Whitefly	480.00	200.00	3.00	98.00	95.00	3.00	0.00	96.94	3.06	100.00	96.94	0.94
Screening	Whitefly	480.00	400.00	1.00	67.00	66.00	0.00	1.00	98.51	0.00	98.51	100.00	1.00
Screening	Whitefly	480.00	400.00	2.00	90.00	82.00	0.00	8.00	91.11	0.00	91.11	100.00	1.00
Screening	Whitefly	480.00	400.00	3.00	85.00	84.00	0.00	1.00	98.82	0.00	98.82	100.00	1.00
Screening	Whitefly	480.00	600.00	1.00	95.00	93.00	1.00	1.00	97.89	1.05	98.95	98.94	0.98
Screening	Whitefly	480.00	600.00	2.00	79.00	74.00	2.00	3.00	93.67	2.53	96.20	97.37	0.95
Screening	Whitefly	480.00	600.00	3.00	67.00	66.00	0.00	1.00	98.51	0.00	98.51	100.00	1.00
Screening	Whitefly	480.00	800.00	1.00	95.00	94.00	1.00	0.00	98.95	1.05	100.00	98.95	0.98
Screening	Whitefly	480.00	800.00	2.00	87.00	83.00	3.00	1.00	95.40	3.45	98.85	96.51	0.93
Screening	Whitefly	480.00	800.00	3.00	48.00	47.00	1.00	0.00	97.92	2.08	100.00	97.92	0.96
Screening	Whitefly	480.00	1000.00	1.00	64.00	56.00	0.00	8.00	87.50	0.00	87.50	100.00	1.00
Screening	Whitefly	480.00	1000.00	2.00	92.00	75.00	0.00	17.00	81.52	0.00	81.52	100.00	1.00
Screening	Whitefly	480.00	1000.00	3.00	72.00	61.00	1.00	10.00	84.72	1.39	86.11	98.39	0.97

Assay type	Species	Wavelength (nm)	Irradiance (μW cm^{-2})	Replicate id	Actual total (n)	Light arm (n)	Dark arm (n)	Base arm (n)	Positive phototaxis (%)	Negative phototaxis (%)	Response rate (%)	Light preference among responders (%)	Choice index among responders
Screening	Whitefly	520.00	100.00	1.00	60.00	36.00	0.00	24.00	60.00	0.00	60.00	100.00	1.00
Screening	Whitefly	520.00	100.00	2.00	46.00	31.00	0.00	15.00	67.39	0.00	67.39	100.00	1.00
Screening	Whitefly	520.00	100.00	3.00	56.00	39.00	0.00	17.00	69.64	0.00	69.64	100.00	1.00
Screening	Whitefly	520.00	200.00	1.00	76.00	72.00	1.00	3.00	94.74	1.32	96.05	98.63	0.97
Screening	Whitefly	520.00	200.00	2.00	86.00	70.00	0.00	16.00	81.40	0.00	81.40	100.00	1.00
Screening	Whitefly	520.00	200.00	3.00	72.00	65.00	0.00	7.00	90.28	0.00	90.28	100.00	1.00
Screening	Whitefly	520.00	400.00	1.00	71.00	42.00	1.00	28.00	59.15	1.41	60.56	97.67	0.95
Screening	Whitefly	520.00	400.00	2.00	40.00	24.00	0.00	16.00	60.00	0.00	60.00	100.00	1.00
Screening	Whitefly	520.00	400.00	3.00	33.00	18.00	0.00	15.00	54.55	0.00	54.55	100.00	1.00
Screening	Whitefly	520.00	600.00	1.00	81.00	67.00	1.00	13.00	82.72	1.23	83.95	98.53	0.97
Screening	Whitefly	520.00	600.00	2.00	106.00	76.00	10.00	20.00	71.70	9.43	81.13	88.37	0.77
Screening	Whitefly	520.00	600.00	3.00	70.00	65.00	5.00	0.00	92.86	7.14	100.00	92.86	0.86
Screening	Whitefly	520.00	800.00	1.00	72.00	65.00	0.00	7.00	90.28	0.00	90.28	100.00	1.00
Screening	Whitefly	520.00	800.00	2.00	66.00	63.00	0.00	3.00	95.45	0.00	95.45	100.00	1.00
Screening	Whitefly	520.00	800.00	3.00	76.00	76.00	0.00	0.00	100.00	0.00	100.00	100.00	1.00
Screening	Whitefly	520.00	1000.00	1.00	70.00	67.00	3.00	0.00	95.71	4.29	100.00	95.71	0.91
Screening	Whitefly	520.00	1000.00	2.00	68.00	64.00	3.00	1.00	94.12	4.41	98.53	95.52	0.91
Screening	Whitefly	520.00	1000.00	3.00	67.00	62.00	3.00	2.00	92.54	4.48	97.01	95.38	0.91
Screening	Whitefly	560.00	100.00	1.00	67.00	32.00	1.00	34.00	47.76	1.49	49.25	96.97	0.94

Assay type	Species	Wavelength (nm)	Irradiance (μW cm^{-2})	Replicate id	Actual total (n)	Light arm (n)	Dark arm (n)	Base arm (n)	Positive phototaxis (%)	Negative phototaxis (%)	Response rate (%)	Light preference among responders (%)	Choice index among responders
Screening	Whitefly	560.00	100.00	2.00	90.00	46.00	1.00	43.00	51.11	1.11	52.22	97.87	0.96
Screening	Whitefly	560.00	100.00	3.00	80.00	36.00	3.00	41.00	45.00	3.75	48.75	92.31	0.85
Screening	Whitefly	560.00	200.00	1.00	86.00	66.00	2.00	18.00	76.74	2.33	79.07	97.06	0.94
Screening	Whitefly	560.00	200.00	2.00	50.00	42.00	3.00	5.00	84.00	6.00	90.00	93.33	0.87
Screening	Whitefly	560.00	200.00	3.00	81.00	59.00	3.00	19.00	72.84	3.70	76.54	95.16	0.90
Screening	Whitefly	560.00	400.00	1.00	101.00	91.00	0.00	10.00	90.10	0.00	90.10	100.00	1.00
Screening	Whitefly	560.00	400.00	2.00	69.00	59.00	0.00	10.00	85.51	0.00	85.51	100.00	1.00
Screening	Whitefly	560.00	400.00	3.00	81.00	70.00	0.00	11.00	86.42	0.00	86.42	100.00	1.00
Screening	Whitefly	560.00	600.00	1.00	72.00	56.00	0.00	16.00	77.78	0.00	77.78	100.00	1.00
Screening	Whitefly	560.00	600.00	2.00	83.00	64.00	0.00	19.00	77.11	0.00	77.11	100.00	1.00
Screening	Whitefly	560.00	600.00	3.00	60.00	46.00	0.00	14.00	76.67	0.00	76.67	100.00	1.00
Screening	Whitefly	560.00	800.00	1.00	75.00	67.00	0.00	8.00	89.33	0.00	89.33	100.00	1.00
Screening	Whitefly	560.00	800.00	2.00	72.00	63.00	0.00	9.00	87.50	0.00	87.50	100.00	1.00
Screening	Whitefly	560.00	800.00	3.00	84.00	81.00	0.00	3.00	96.43	0.00	96.43	100.00	1.00
Screening	Whitefly	560.00	1000.00	1.00	112.00	57.00	2.00	53.00	50.89	1.79	52.68	96.61	0.93
Screening	Whitefly	560.00	1000.00	2.00	70.00	39.00	3.00	28.00	55.71	4.29	60.00	92.86	0.86
Screening	Whitefly	560.00	1000.00	3.00	83.00	47.00	2.00	34.00	56.63	2.41	59.04	95.92	0.92
Screening	Whitefly	600.00	100.00	1.00	98.00	65.00	3.00	30.00	66.33	3.06	69.39	95.59	0.91
Screening	Whitefly	600.00	100.00	2.00	61.00	42.00	5.00	14.00	68.85	8.20	77.05	89.36	0.79

Assay type	Species	Wavelength (nm)	Irradiance (μW cm^{-2})	Replicate id	Actual total (n)	Light arm (n)	Dark arm (n)	Base arm (n)	Positive phototaxis (%)	Negative phototaxis (%)	Response rate (%)	Light preference among responders (%)	Choice index among responders
Screening	Whitefly	600.00	100.00	3.00	79.00	57.00	3.00	19.00	72.15	3.80	75.95	95.00	0.90
Screening	Whitefly	600.00	200.00	1.00	94.00	75.00	5.00	14.00	79.79	5.32	85.11	93.75	0.88
Screening	Whitefly	600.00	200.00	2.00	88.00	71.00	0.00	17.00	80.68	0.00	80.68	100.00	1.00
Screening	Whitefly	600.00	200.00	3.00	93.00	77.00	4.00	12.00	82.80	4.30	87.10	95.06	0.90
Screening	Whitefly	600.00	400.00	1.00	74.00	55.00	0.00	19.00	74.32	0.00	74.32	100.00	1.00
Screening	Whitefly	600.00	400.00	2.00	65.00	49.00	1.00	15.00	75.38	1.54	76.92	98.00	0.96
Screening	Whitefly	600.00	400.00	3.00	76.00	59.00	1.00	16.00	77.63	1.32	78.95	98.33	0.97
Screening	Whitefly	600.00	600.00	1.00	105.00	76.00	0.00	29.00	72.38	0.00	72.38	100.00	1.00
Screening	Whitefly	600.00	600.00	2.00	75.00	58.00	0.00	17.00	77.33	0.00	77.33	100.00	1.00
Screening	Whitefly	600.00	600.00	3.00	72.00	56.00	0.00	16.00	77.78	0.00	77.78	100.00	1.00
Screening	Whitefly	600.00	800.00	1.00	80.00	61.00	2.00	17.00	76.25	2.50	78.75	96.83	0.94
Screening	Whitefly	600.00	800.00	2.00	79.00	56.00	2.00	21.00	70.89	2.53	73.42	96.55	0.93
Screening	Whitefly	600.00	800.00	3.00	70.00	52.00	3.00	15.00	74.29	4.29	78.57	94.55	0.89
Screening	Whitefly	600.00	1000.00	1.00	70.00	60.00	3.00	7.00	85.71	4.29	90.00	95.24	0.90
Screening	Whitefly	600.00	1000.00	2.00	80.00	69.00	5.00	6.00	86.25	6.25	92.50	93.24	0.86
Screening	Whitefly	600.00	1000.00	3.00	88.00	76.00	3.00	9.00	86.36	3.41	89.77	96.20	0.92
Screening	Whitefly	640.00	100.00	1.00	57.00	35.00	3.00	19.00	61.40	5.26	66.67	92.11	0.84
Screening	Whitefly	640.00	100.00	2.00	66.00	33.00	8.00	25.00	50.00	12.12	62.12	80.49	0.61
Screening	Whitefly	640.00	100.00	3.00	89.00	51.00	13.00	25.00	57.30	14.61	71.91	79.69	0.59

Assay type	Species	Wavelength (nm)	Irradiance (μW cm⁻²)	Replicate id	Actual total (n)	Light arm (n)	Dark arm (n)	Base arm (n)	Positive phototaxis (%)	Negative phototaxis (%)	Response rate (%)	Light preference among responders (%)	Choice index among responders
Screening	Whitefly	640.00	200.00	1.00	76.00	57.00	2.00	17.00	75.00	2.63	77.63	96.61	0.93
Screening	Whitefly	640.00	200.00	2.00	80.00	65.00	3.00	12.00	81.25	3.75	85.00	95.59	0.91
Screening	Whitefly	640.00	200.00	3.00	82.00	69.00	5.00	8.00	84.15	6.10	90.24	93.24	0.86
Screening	Whitefly	640.00	400.00	1.00	76.00	51.00	1.00	24.00	67.11	1.32	68.42	98.08	0.96
Screening	Whitefly	640.00	400.00	2.00	65.00	45.00	0.00	20.00	69.23	0.00	69.23	100.00	1.00
Screening	Whitefly	640.00	400.00	3.00	65.00	43.00	1.00	21.00	66.15	1.54	67.69	97.73	0.95
Screening	Whitefly	640.00	600.00	1.00	88.00	47.00	11.00	30.00	53.41	12.50	65.91	81.03	0.62
Screening	Whitefly	640.00	600.00	2.00	77.00	44.00	21.00	12.00	57.14	27.27	84.42	67.69	0.35
Screening	Whitefly	640.00	600.00	3.00	80.00	44.00	6.00	30.00	55.00	7.50	62.50	88.00	0.76
Screening	Whitefly	640.00	800.00	1.00	68.00	54.00	4.00	10.00	79.41	5.88	85.29	93.10	0.86
Screening	Whitefly	640.00	800.00	2.00	50.00	40.00	3.00	7.00	80.00	6.00	86.00	93.02	0.86
Screening	Whitefly	640.00	800.00	3.00	65.00	57.00	1.00	7.00	87.69	1.54	89.23	98.28	0.97
Screening	Whitefly	640.00	1000.00	1.00	68.00	60.00	0.00	8.00	88.24	0.00	88.24	100.00	1.00
Screening	Whitefly	640.00	1000.00	2.00	88.00	78.00	5.00	5.00	88.64	5.68	94.32	93.98	0.88
Screening	Whitefly	640.00	1000.00	3.00	65.00	59.00	2.00	4.00	90.77	3.08	93.85	96.72	0.93
Screening	Predator	440.00	100.00	1.00	36.00	6.00	9.00	21.00	16.67	25.00	41.67	40.00	-0.20
Screening	Predator	440.00	100.00	2.00	34.00	8.00	11.00	15.00	23.53	32.35	55.88	42.11	-0.16
Screening	Predator	440.00	100.00	3.00	34.00	6.00	12.00	16.00	17.65	35.29	52.94	33.33	-0.33
Screening	Predator	440.00	200.00	1.00	40.00	7.00	17.00	16.00	17.50	42.50	60.00	29.17	-0.42

Assay type	Species	Wavelength (nm)	Irradiance (μW cm⁻²)	Replicate id	Actual total (n)	Light arm (n)	Dark arm (n)	Base arm (n)	Positive phototaxis (%)	Negative phototaxis (%)	Response rate (%)	Light preference among responders (%)	Choice index among responders
Screening	Predator	440.00	200.00	2.00	43.00	5.00	22.00	16.00	11.63	51.16	62.79	18.52	-0.63
Screening	Predator	440.00	200.00	3.00	48.00	7.00	19.00	22.00	14.58	39.58	54.17	26.92	-0.46
Screening	Predator	440.00	400.00	1.00	33.00	6.00	15.00	12.00	18.18	45.45	63.64	28.57	-0.43
Screening	Predator	440.00	400.00	2.00	35.00	7.00	15.00	13.00	20.00	42.86	62.86	31.82	-0.36
Screening	Predator	440.00	400.00	3.00	36.00	5.00	15.00	16.00	13.89	41.67	55.56	25.00	-0.50
Screening	Predator	440.00	600.00	1.00	41.00	8.00	17.00	16.00	19.51	41.46	60.98	32.00	-0.36
Screening	Predator	440.00	600.00	2.00	41.00	8.00	15.00	18.00	19.51	36.59	56.10	34.78	-0.30
Screening	Predator	440.00	600.00	3.00	46.00	10.00	20.00	16.00	21.74	43.48	65.22	33.33	-0.33
Screening	Predator	440.00	800.00	1.00	32.00	7.00	15.00	10.00	21.88	46.88	68.75	31.82	-0.36
Screening	Predator	440.00	800.00	2.00	37.00	8.00	15.00	14.00	21.62	40.54	62.16	34.78	-0.30
Screening	Predator	440.00	800.00	3.00	41.00	12.00	17.00	12.00	29.27	41.46	70.73	41.38	-0.17
Screening	Predator	440.00	1000.00	1.00	36.00	6.00	13.00	17.00	16.67	36.11	52.78	31.58	-0.37
Screening	Predator	440.00	1000.00	2.00	40.00	8.00	14.00	18.00	20.00	35.00	55.00	36.36	-0.27
Screening	Predator	440.00	1000.00	3.00	44.00	6.00	20.00	18.00	13.64	45.45	59.09	23.08	-0.54
Screening	Predator	400.00	100.00	1.00	39.00	9.00	16.00	14.00	23.08	41.03	64.10	36.00	-0.28
Screening	Predator	400.00	100.00	2.00	33.00	7.00	16.00	10.00	21.21	48.48	69.70	30.43	-0.39
Screening	Predator	400.00	100.00	3.00	37.00	11.00	19.00	7.00	29.73	51.35	81.08	36.67	-0.27
Screening	Predator	400.00	200.00	1.00	39.00	6.00	17.00	16.00	15.38	43.59	58.97	26.09	-0.48
Screening	Predator	400.00	200.00	2.00	41.00	8.00	15.00	18.00	19.51	36.59	56.10	34.78	-0.30

Assay type	Species	Wavelength (nm)	Irradiance (μW cm⁻²)	Replicate id	Actual total (n)	Light arm (n)	Dark arm (n)	Base arm (n)	Positive phototaxis (%)	Negative phototaxis (%)	Response rate (%)	Light preference among responders (%)	Choice index among responders
Screening	Predator	400.00	200.00	3.00	41.00	9.00	17.00	15.00	21.95	41.46	63.41	34.62	-0.31
Screening	Predator	400.00	400.00	1.00	43.00	15.00	19.00	9.00	34.88	44.19	79.07	44.12	-0.12
Screening	Predator	400.00	400.00	2.00	28.00	8.00	15.00	5.00	28.57	53.57	82.14	34.78	-0.30
Screening	Predator	400.00	400.00	3.00	33.00	11.00	16.00	6.00	33.33	48.48	81.82	40.74	-0.19
Screening	Predator	400.00	600.00	1.00	36.00	6.00	18.00	12.00	16.67	50.00	66.67	25.00	-0.50
Screening	Predator	400.00	600.00	2.00	35.00	6.00	20.00	9.00	17.14	57.14	74.29	23.08	-0.54
Screening	Predator	400.00	600.00	3.00	28.00	3.00	18.00	7.00	10.71	64.29	75.00	14.29	-0.71
Screening	Predator	400.00	800.00	1.00	40.00	7.00	18.00	15.00	17.50	45.00	62.50	28.00	-0.44
Screening	Predator	400.00	800.00	2.00	41.00	7.00	22.00	12.00	17.07	53.66	70.73	24.14	-0.52
Screening	Predator	400.00	800.00	3.00	41.00	8.00	23.00	10.00	19.51	56.10	75.61	25.81	-0.48
Screening	Predator	400.00	1000.00	1.00	30.00	4.00	16.00	10.00	13.33	53.33	66.67	20.00	-0.60
Screening	Predator	400.00	1000.00	2.00	34.00	5.00	16.00	13.00	14.71	47.06	61.76	23.81	-0.52
Screening	Predator	400.00	1000.00	3.00	38.00	7.00	17.00	14.00	18.42	44.74	63.16	29.17	-0.42
Screening	Predator	480.00	100.00	1.00	45.00	15.00	6.00	24.00	33.33	13.33	46.67	71.43	0.43
Screening	Predator	480.00	100.00	2.00	41.00	11.00	8.00	22.00	26.83	19.51	46.34	57.89	0.16
Screening	Predator	480.00	100.00	3.00	36.00	8.00	8.00	20.00	22.22	22.22	44.44	50.00	0.00
Screening	Predator	480.00	200.00	1.00	35.00	12.00	8.00	15.00	34.29	22.86	57.14	60.00	0.20
Screening	Predator	480.00	200.00	2.00	48.00	15.00	10.00	23.00	31.25	20.83	52.08	60.00	0.20
Screening	Predator	480.00	200.00	3.00	43.00	17.00	6.00	20.00	39.53	13.95	53.49	73.91	0.48

Assay type	Species	Wavelength (nm)	Irradiance (µW cm^{-2})	Replicate id	Actual total (n)	Light arm (n)	Dark arm (n)	Base arm (n)	Positive phototaxis (%)	Negative phototaxis (%)	Response rate (%)	Light preference among responders (%)	Choice index among responders
Screening	Predator	480.00	400.00	1.00	40.00	8.00	12.00	20.00	20.00	30.00	50.00	40.00	-0.20
Screening	Predator	480.00	400.00	2.00	43.00	11.00	11.00	21.00	25.58	25.58	51.16	50.00	0.00
Screening	Predator	480.00	400.00	3.00	34.00	8.00	12.00	14.00	23.53	35.29	58.82	40.00	-0.20
Screening	Predator	480.00	600.00	1.00	43.00	19.00	5.00	19.00	44.19	11.63	55.81	79.17	0.58
Screening	Predator	480.00	600.00	2.00	37.00	15.00	9.00	13.00	40.54	24.32	64.86	62.50	0.25
Screening	Predator	480.00	600.00	3.00	41.00	18.00	4.00	19.00	43.90	9.76	53.66	81.82	0.64
Screening	Predator	480.00	800.00	1.00	39.00	16.00	6.00	17.00	41.03	15.38	56.41	72.73	0.45
Screening	Predator	480.00	800.00	2.00	43.00	14.00	9.00	20.00	32.56	20.93	53.49	60.87	0.22
Screening	Predator	480.00	800.00	3.00	45.00	16.00	3.00	26.00	35.56	6.67	42.22	84.21	0.68
Screening	Predator	480.00	1000.00	1.00	43.00	17.00	10.00	16.00	39.53	23.26	62.79	62.96	0.26
Screening	Predator	480.00	1000.00	2.00	40.00	15.00	10.00	15.00	37.50	25.00	62.50	60.00	0.20
Screening	Predator	480.00	1000.00	3.00	42.00	20.00	10.00	12.00	47.62	23.81	71.43	66.67	0.33
Screening	Predator	560.00	100.00	1.00	46.00	18.00	8.00	20.00	39.13	17.39	56.52	69.23	0.38
Screening	Predator	560.00	100.00	2.00	39.00	15.00	7.00	17.00	38.46	17.95	56.41	68.18	0.36
Screening	Predator	560.00	100.00	3.00	41.00	12.00	7.00	22.00	29.27	17.07	46.34	63.16	0.26
Screening	Predator	560.00	200.00	1.00	43.00	13.00	8.00	22.00	30.23	18.60	48.84	61.90	0.24
Screening	Predator	560.00	200.00	2.00	45.00	16.00	8.00	21.00	35.56	17.78	53.33	66.67	0.33
Screening	Predator	560.00	200.00	3.00	38.00	9.00	9.00	20.00	23.68	23.68	47.37	50.00	0.00
Screening	Predator	560.00	400.00	1.00	47.00	12.00	11.00	24.00	25.53	23.40	48.94	52.17	0.04

Assay type	Species	Wavelength (nm)	Irradiance ($\mu W\ cm^{-2}$)	Replicate id	Actual total (n)	Light arm (n)	Dark arm (n)	Base arm (n)	Positive phototaxis (%)	Negative phototaxis (%)	Response rate (%)	Light preference among responders (%)	Choice index among responders
Screening	Predator	560.00	400.00	2.00	46.00	11.00	9.00	26.00	23.91	19.57	43.48	55.00	0.10
Screening	Predator	560.00	400.00	3.00	47.00	11.00	8.00	28.00	23.40	17.02	40.43	57.89	0.16
Screening	Predator	560.00	600.00	1.00	46.00	10.00	8.00	28.00	21.74	17.39	39.13	55.56	0.11
Screening	Predator	560.00	600.00	2.00	46.00	11.00	8.00	27.00	23.91	17.39	41.30	57.89	0.16
Screening	Predator	560.00	600.00	3.00	29.00	8.00	6.00	15.00	27.59	20.69	48.28	57.14	0.14
Screening	Predator	560.00	800.00	1.00	46.00	10.00	8.00	28.00	21.74	17.39	39.13	55.56	0.11
Screening	Predator	560.00	800.00	2.00	48.00	12.00	8.00	28.00	25.00	16.67	41.67	60.00	0.20
Screening	Predator	560.00	800.00	3.00	44.00	10.00	9.00	25.00	22.73	20.45	43.18	52.63	0.05
Screening	Predator	560.00	1000.00	1.00	41.00	14.00	13.00	14.00	34.15	31.71	65.85	51.85	0.04
Screening	Predator	560.00	1000.00	2.00	46.00	11.00	11.00	24.00	23.91	23.91	47.83	50.00	0.00
Screening	Predator	560.00	1000.00	3.00	48.00	18.00	12.00	18.00	37.50	25.00	62.50	60.00	0.20
Screening	Predator	600.00	100.00	1.00	48.00	16.00	10.00	22.00	33.33	20.83	54.17	61.54	0.23
Screening	Predator	600.00	100.00	2.00	48.00	15.00	12.00	21.00	31.25	25.00	56.25	55.56	0.11
Screening	Predator	600.00	100.00	3.00	49.00	18.00	10.00	21.00	36.73	20.41	57.14	64.29	0.29
Screening	Predator	600.00	200.00	1.00	50.00	18.00	9.00	23.00	36.00	18.00	54.00	66.67	0.33
Screening	Predator	600.00	200.00	2.00	43.00	15.00	8.00	20.00	34.88	18.60	53.49	65.22	0.30
Screening	Predator	600.00	200.00	3.00	45.00	17.00	8.00	20.00	37.78	17.78	55.56	68.00	0.36
Screening	Predator	600.00	400.00	1.00	50.00	20.00	9.00	21.00	40.00	18.00	58.00	68.97	0.38
Screening	Predator	600.00	400.00	2.00	50.00	22.00	8.00	20.00	44.00	16.00	60.00	73.33	0.47

Assay type	Species	Wavelength (nm)	Irradiance (µW cm^{-2})	Replicate id	Actual total (n)	Light arm (n)	Dark arm (n)	Base arm (n)	Positive phototaxis (%)	Negative phototaxis (%)	Response rate (%)	Light preference among responders (%)	Choice index among responders
Screening	Predator	600.00	400.00	3.00	54.00	20.00	12.00	22.00	37.04	22.22	59.26	62.50	0.25
Screening	Predator	600.00	600.00	1.00	50.00	16.00	11.00	23.00	32.00	22.00	54.00	59.26	0.19
Screening	Predator	600.00	600.00	2.00	48.00	13.00	9.00	26.00	27.08	18.75	45.83	59.09	0.18
Screening	Predator	600.00	600.00	3.00	50.00	17.00	8.00	25.00	34.00	16.00	50.00	68.00	0.36
Screening	Predator	600.00	800.00	1.00	47.00	17.00	10.00	20.00	36.17	21.28	57.45	62.96	0.26
Screening	Predator	600.00	800.00	2.00	45.00	15.00	12.00	18.00	33.33	26.67	60.00	55.56	0.11
Screening	Predator	600.00	800.00	3.00	50.00	15.00	13.00	22.00	30.00	26.00	56.00	53.57	0.07
Screening	Predator	600.00	1000.00	1.00	43.00	14.00	11.00	18.00	32.56	25.58	58.14	56.00	0.12
Screening	Predator	600.00	1000.00	2.00	50.00	15.00	12.00	23.00	30.00	24.00	54.00	55.56	0.11
Screening	Predator	600.00	1000.00	3.00	48.00	17.00	10.00	21.00	35.42	20.83	56.25	62.96	0.26
Screening	Predator	640.00	100.00	1.00	47.00	19.00	8.00	20.00	40.43	17.02	57.45	70.37	0.41
Screening	Predator	640.00	100.00	2.00	43.00	19.00	12.00	12.00	44.19	27.91	72.09	61.29	0.23
Screening	Predator	640.00	100.00	3.00	46.00	20.00	12.00	14.00	43.48	26.09	69.57	62.50	0.25
Screening	Predator	640.00	200.00	1.00	32.00	13.00	3.00	16.00	40.62	9.38	50.00	81.25	0.62
Screening	Predator	640.00	200.00	2.00	41.00	17.00	8.00	16.00	41.46	19.51	60.98	68.00	0.36
Screening	Predator	640.00	200.00	3.00	43.00	13.00	7.00	23.00	30.23	16.28	46.51	65.00	0.30
Screening	Predator	640.00	400.00	1.00	39.00	14.00	10.00	15.00	35.90	25.64	61.54	58.33	0.17
Screening	Predator	640.00	400.00	2.00	38.00	12.00	11.00	15.00	31.58	28.95	60.53	52.17	0.04
Screening	Predator	640.00	400.00	3.00	34.00	10.00	11.00	13.00	29.41	32.35	61.76	47.62	-0.05

Assay type	Species	Wavelength (nm)	Irradiance (μW cm^{-2})	Replicate id	Actual total (n)	Light arm (n)	Dark arm (n)	Base arm (n)	Positive phototaxis (%)	Negative phototaxis (%)	Response rate (%)	Light preference among responders (%)	Choice index among responders
Screening	Predator	640.00	600.00	1.00	44.00	16.00	3.00	25.00	36.36	6.82	43.18	84.21	0.68
Screening	Predator	640.00	600.00	2.00	39.00	14.00	4.00	21.00	35.90	10.26	46.15	77.78	0.56
Screening	Predator	640.00	600.00	3.00	42.00	16.00	3.00	23.00	38.10	7.14	45.24	84.21	0.68
Screening	Predator	640.00	800.00	1.00	38.00	15.00	5.00	18.00	39.47	13.16	52.63	75.00	0.50
Screening	Predator	640.00	800.00	2.00	42.00	14.00	6.00	22.00	33.33	14.29	47.62	70.00	0.40
Screening	Predator	640.00	800.00	3.00	44.00	13.00	6.00	25.00	29.55	13.64	43.18	68.42	0.37
Screening	Predator	640.00	1000.00	1.00	34.00	10.00	7.00	17.00	29.41	20.59	50.00	58.82	0.18
Screening	Predator	640.00	1000.00	2.00	40.00	10.00	8.00	22.00	25.00	20.00	45.00	55.56	0.11
Screening	Predator	640.00	1000.00	3.00	41.00	10.00	10.00	21.00	24.39	24.39	48.78	50.00	0.00
Screening	Predator	520.00	100.00	1.00	47.00	12.00	10.00	25.00	25.53	21.28	46.81	54.55	0.09
Screening	Predator	520.00	100.00	2.00	42.00	9.00	13.00	20.00	21.43	30.95	52.38	40.91	-0.18
Screening	Predator	520.00	100.00	3.00	45.00	8.00	12.00	25.00	17.78	26.67	44.44	40.00	-0.20
Screening	Predator	520.00	200.00	1.00	46.00	16.00	10.00	20.00	34.78	21.74	56.52	61.54	0.23
Screening	Predator	520.00	200.00	2.00	48.00	16.00	7.00	25.00	33.33	14.58	47.92	69.57	0.39
Screening	Predator	520.00	200.00	3.00	49.00	15.00	8.00	26.00	30.61	16.33	46.94	65.22	0.30
Screening	Predator	520.00	400.00	1.00	48.00	15.00	8.00	25.00	31.25	16.67	47.92	65.22	0.30
Screening	Predator	520.00	400.00	2.00	46.00	18.00	9.00	19.00	39.13	19.57	58.70	66.67	0.33
Screening	Predator	520.00	400.00	3.00	44.00	16.00	10.00	18.00	36.36	22.73	59.09	61.54	0.23
Screening	Predator	520.00	600.00	1.00	40.00	15.00	8.00	17.00	37.50	20.00	57.50	65.22	0.30

Assay type	Species	Wavelength (nm)	Irradiance (μW cm^{-2})	Replicate id	Actual total (n)	Light arm (n)	Dark arm (n)	Base arm (n)	Positive phototaxis (%)	Negative phototaxis (%)	Response rate (%)	Light preference among responders (%)	Choice index among responders
Screening	Predator	520.00	600.00	2.00	41.00	12.00	10.00	19.00	29.27	24.39	53.66	54.55	0.09
Screening	Predator	520.00	600.00	3.00	49.00	16.00	10.00	23.00	32.65	20.41	53.06	61.54	0.23
Screening	Predator	520.00	800.00	1.00	44.00	13.00	11.00	20.00	29.55	25.00	54.55	54.17	0.08
Screening	Predator	520.00	800.00	2.00	45.00	15.00	10.00	20.00	33.33	22.22	55.56	60.00	0.20
Screening	Predator	520.00	800.00	3.00	47.00	15.00	12.00	20.00	31.91	25.53	57.45	55.56	0.11
Screening	Predator	520.00	1000.00	1.00	45.00	19.00	10.00	16.00	42.22	22.22	64.44	65.52	0.31
Screening	Predator	520.00	1000.00	2.00	45.00	15.00	10.00	20.00	33.33	22.22	55.56	60.00	0.20
Screening	Predator	520.00	1000.00	3.00	44.00	16.00	10.00	18.00	36.36	22.73	59.09	61.54	0.23

Table S2. Replicate-level temperature-assay counts and derived phototaxis metrics for both species.

Assay type	Species	Wavelength (nm)	Irradiance ($\mu W\ cm^{-2}$)	Temperature (°C)	Replicate id	Actual total (n)	Light arm (n)	Dark arm (n)	Base arm (n)	Positive phototaxis (%)	Negative phototaxis (%)	Response rate (%)	Light preference among responders (%)	Choice index among responders
Temperature	Predator	400.00	600.00	20.00	1.00	30.00	2.00	25.00	3.00	6.67	83.33	90.00	7.41	-0.85
Temperature	Predator	400.00	600.00	20.00	2.00	29.00	0.00	28.00	1.00	0.00	96.55	96.55	0.00	-1.00
Temperature	Predator	400.00	600.00	20.00	3.00	25.00	1.00	23.00	1.00	4.00	92.00	96.00	4.17	-0.92
Temperature	Predator	400.00	600.00	25.00	1.00	30.00	10.00	14.00	6.00	33.33	46.67	80.00	41.67	-0.17
Temperature	Predator	400.00	600.00	25.00	2.00	22.00	4.00	14.00	4.00	18.18	63.64	81.82	22.22	-0.56
Temperature	Predator	400.00	600.00	25.00	3.00	28.00	7.00	16.00	5.00	25.00	57.14	82.14	30.43	-0.39
Temperature	Predator	400.00	600.00	30.00	1.00	29.00	10.00	14.00	5.00	34.48	48.28	82.76	41.67	-0.17
Temperature	Predator	400.00	600.00	30.00	2.00	28.00	9.00	12.00	7.00	32.14	42.86	75.00	42.86	-0.14
Temperature	Predator	400.00	600.00	30.00	3.00	28.00	9.00	10.00	9.00	32.14	35.71	67.86	47.37	-0.05
Temperature	Predator	400.00	600.00	35.00	1.00	27.00	6.00	5.00	16.00	22.22	18.52	40.74	54.55	0.09
Temperature	Predator	400.00	600.00	35.00	2.00	28.00	8.00	5.00	15.00	28.57	17.86	46.43	61.54	0.23
Temperature	Predator	400.00	600.00	35.00	3.00	29.00	8.00	6.00	15.00	27.59	20.69	48.28	57.14	0.14
Temperature	Predator	440.00	600.00	20.00	1.00	29.00	0.00	20.00	9.00	0.00	68.97	68.97	0.00	-1.00
Temperature	Predator	440.00	600.00	20.00	2.00	26.00	0.00	16.00	10.00	0.00	61.54	61.54	0.00	-1.00
Temperature	Predator	440.00	600.00	20.00	3.00	23.00	1.00	15.00	7.00	4.35	65.22	69.57	6.25	-0.88
Temperature	Predator	440.00	600.00	25.00	1.00	27.00	10.00	12.00	5.00	37.04	44.44	81.48	45.45	-0.09
Temperature	Predator	440.00	600.00	25.00	2.00	26.00	8.00	14.00	4.00	30.77	53.85	84.62	36.36	-0.27

Temperature	Predator	440.00	600.00	25.00	3.00	27.00	11.00	12.00	4.00	40.74	44.44	85.19	47.83	-0.04
Temperature	Predator	440.00	600.00	30.00	1.00	30.00	14.00	8.00	8.00	46.67	26.67	73.33	63.64	0.27
Temperature	Predator	440.00	600.00	30.00	2.00	26.00	11.00	5.00	10.00	42.31	19.23	61.54	68.75	0.38
Temperature	Predator	440.00	600.00	30.00	3.00	28.00	13.00	6.00	9.00	46.43	21.43	67.86	68.42	0.37
Temperature	Predator	440.00	600.00	35.00	1.00	28.00	6.00	8.00	14.00	21.43	28.57	50.00	42.86	-0.14
Temperature	Predator	440.00	600.00	35.00	2.00	28.00	4.00	8.00	16.00	14.29	28.57	42.86	33.33	-0.33
Temperature	Predator	440.00	600.00	35.00	3.00	22.00	4.00	4.00	14.00	18.18	18.18	36.36	50.00	0.00
Temperature	Predator	480.00	400.00	20.00	1.00	30.00	8.00	15.00	7.00	26.67	50.00	76.67	34.78	-0.30
Temperature	Predator	480.00	400.00	20.00	2.00	30.00	7.00	18.00	5.00	23.33	60.00	83.33	28.00	-0.44
Temperature	Predator	480.00	400.00	20.00	3.00	23.00	5.00	13.00	5.00	21.74	56.52	78.26	27.78	-0.44
Temperature	Predator	480.00	400.00	25.00	1.00	28.00	10.00	7.00	11.00	35.71	25.00	60.71	58.82	0.18
Temperature	Predator	480.00	400.00	25.00	2.00	26.00	12.00	7.00	7.00	46.15	26.92	73.08	63.16	0.26
Temperature	Predator	480.00	400.00	25.00	3.00	28.00	12.00	6.00	10.00	42.86	21.43	64.29	66.67	0.33
Temperature	Predator	480.00	400.00	30.00	1.00	27.00	13.00	10.00	4.00	48.15	37.04	85.19	56.52	0.13
Temperature	Predator	480.00	400.00	30.00	2.00	26.00	10.00	8.00	8.00	38.46	30.77	69.23	55.56	0.11
Temperature	Predator	480.00	400.00	30.00	3.00	27.00	12.00	9.00	6.00	44.44	33.33	77.78	57.14	0.14
Temperature	Predator	480.00	400.00	35.00	1.00	27.00	13.00	5.00	9.00	48.15	18.52	66.67	72.22	0.44
Temperature	Predator	480.00	400.00	35.00	2.00	28.00	10.00	8.00	10.00	35.71	28.57	64.29	55.56	0.11
Temperature	Predator	480.00	400.00	35.00	3.00	29.00	11.00	8.00	10.00	37.93	27.59	65.52	57.89	0.16
Temperature	Predator	480.00	800.00	20.00	1.00	29.00	4.00	15.00	10.00	13.79	51.72	65.52	21.05	-0.58
Temperature	Predator	480.00	800.00	20.00	2.00	28.00	4.00	14.00	10.00	14.29	50.00	64.29	22.22	-0.56

Temperature	Predator	480.00	800.00	20.00	3.00	29.00	9.00	15.00	5.00	31.03	51.72	82.76	37.50	-0.25
Temperature	Predator	480.00	800.00	25.00	1.00	28.00	10.00	10.00	8.00	35.71	35.71	71.43	50.00	0.00
Temperature	Predator	480.00	800.00	25.00	2.00	28.00	6.00	8.00	14.00	21.43	28.57	50.00	42.86	-0.14
Temperature	Predator	480.00	800.00	25.00	3.00	28.00	8.00	10.00	10.00	28.57	35.71	64.29	44.44	-0.11
Temperature	Predator	480.00	800.00	30.00	1.00	30.00	16.00	5.00	9.00	53.33	16.67	70.00	76.19	0.52
Temperature	Predator	480.00	800.00	30.00	2.00	25.00	12.00	8.00	5.00	48.00	32.00	80.00	60.00	0.20
Temperature	Predator	480.00	800.00	30.00	3.00	27.00	14.00	6.00	7.00	51.85	22.22	74.07	70.00	0.40
Temperature	Predator	480.00	800.00	35.00	1.00	28.00	19.00	4.00	5.00	67.86	14.29	82.14	82.61	0.65
Temperature	Predator	480.00	800.00	35.00	2.00	29.00	14.00	6.00	9.00	48.28	20.69	68.97	70.00	0.40
Temperature	Predator	480.00	800.00	35.00	3.00	28.00	15.00	5.00	8.00	53.57	17.86	71.43	75.00	0.50
Temperature	Predator	520.00	400.00	20.00	1.00	26.00	10.00	4.00	12.00	38.46	15.38	53.85	71.43	0.43
Temperature	Predator	520.00	400.00	20.00	2.00	25.00	7.00	6.00	12.00	28.00	24.00	52.00	53.85	0.08
Temperature	Predator	520.00	400.00	20.00	3.00	26.00	8.00	8.00	10.00	30.77	30.77	61.54	50.00	0.00
Temperature	Predator	520.00	400.00	25.00	1.00	30.00	13.00	3.00	14.00	43.33	10.00	53.33	81.25	0.62
Temperature	Predator	520.00	400.00	25.00	2.00	25.00	12.00	4.00	9.00	48.00	16.00	64.00	75.00	0.50
Temperature	Predator	520.00	400.00	25.00	3.00	27.00	12.00	5.00	10.00	44.44	18.52	62.96	70.59	0.41
Temperature	Predator	520.00	400.00	30.00	1.00	30.00	18.00	3.00	9.00	60.00	10.00	70.00	85.71	0.71
Temperature	Predator	520.00	400.00	30.00	2.00	29.00	15.00	4.00	10.00	51.72	13.79	65.52	78.95	0.58
Temperature	Predator	520.00	400.00	30.00	3.00	30.00	16.00	4.00	10.00	53.33	13.33	66.67	80.00	0.60
Temperature	Predator	520.00	400.00	35.00	1.00	25.00	4.00	9.00	12.00	16.00	36.00	52.00	30.77	-0.38
Temperature	Predator	520.00	400.00	35.00	2.00	25.00	6.00	6.00	13.00	24.00	24.00	48.00	50.00	0.00

Temperature	Predator	520.00	400.00	35.00	3.00	26.00	6.00	8.00	12.00	23.08	30.77	53.85	42.86	-0.14
Temperature	Predator	520.00	800.00	20.00	1.00	25.00	5.00	10.00	10.00	20.00	40.00	60.00	33.33	-0.33
Temperature	Predator	520.00	800.00	20.00	2.00	24.00	4.00	8.00	12.00	16.67	33.33	50.00	33.33	-0.33
Temperature	Predator	520.00	800.00	20.00	3.00	28.00	4.00	12.00	12.00	14.29	42.86	57.14	25.00	-0.50
Temperature	Predator	520.00	800.00	25.00	1.00	30.00	16.00	5.00	9.00	53.33	16.67	70.00	76.19	0.52
Temperature	Predator	520.00	800.00	25.00	2.00	30.00	14.00	8.00	8.00	46.67	26.67	73.33	63.64	0.27
Temperature	Predator	520.00	800.00	25.00	3.00	34.00	15.00	9.00	10.00	44.12	26.47	70.59	62.50	0.25
Temperature	Predator	520.00	800.00	30.00	1.00	28.00	12.00	8.00	8.00	42.86	28.57	71.43	60.00	0.20
Temperature	Predator	520.00	800.00	30.00	2.00	28.00	10.00	8.00	10.00	35.71	28.57	64.29	55.56	0.11
Temperature	Predator	520.00	800.00	30.00	3.00	26.00	12.00	4.00	10.00	46.15	15.38	61.54	75.00	0.50
Temperature	Predator	520.00	800.00	35.00	1.00	29.00	12.00	8.00	9.00	41.38	27.59	68.97	60.00	0.20
Temperature	Predator	520.00	800.00	35.00	2.00	30.00	17.00	6.00	7.00	56.67	20.00	76.67	73.91	0.48
Temperature	Predator	520.00	800.00	35.00	3.00	29.00	15.00	5.00	9.00	51.72	17.24	68.97	75.00	0.50
Temperature	Predator	600.00	1000.00	20.00	1.00	26.00	8.00	10.00	8.00	30.77	38.46	69.23	44.44	-0.11
Temperature	Predator	600.00	1000.00	20.00	2.00	30.00	9.00	12.00	9.00	30.00	40.00	70.00	42.86	-0.14
Temperature	Predator	600.00	1000.00	20.00	3.00	26.00	8.00	12.00	6.00	30.77	46.15	76.92	40.00	-0.20
Temperature	Predator	600.00	1000.00	25.00	1.00	28.00	8.00	12.00	8.00	28.57	42.86	71.43	40.00	-0.20
Temperature	Predator	600.00	1000.00	25.00	2.00	27.00	12.00	10.00	5.00	44.44	37.04	81.48	54.55	0.09
Temperature	Predator	600.00	1000.00	25.00	3.00	28.00	10.00	11.00	7.00	35.71	39.29	75.00	47.62	-0.05
Temperature	Predator	600.00	1000.00	30.00	1.00	30.00	14.00	9.00	7.00	46.67	30.00	76.67	60.87	0.22
Temperature	Predator	600.00	1000.00	30.00	2.00	30.00	14.00	11.00	5.00	46.67	36.67	83.33	56.00	0.12

Temperature	Predator	600.00	1000.00	30.00	3.00	29.00	13.00	10.00	6.00	44.83	34.48	79.31	56.52	0.13
Temperature	Predator	600.00	1000.00	35.00	1.00	30.00	10.00	9.00	11.00	33.33	30.00	63.33	52.63	0.05
Temperature	Predator	600.00	1000.00	35.00	2.00	27.00	8.00	9.00	10.00	29.63	33.33	62.96	47.06	-0.06
Temperature	Predator	600.00	1000.00	35.00	3.00	28.00	8.00	8.00	12.00	28.57	28.57	57.14	50.00	0.00
Temperature	Predator	640.00	1000.00	20.00	1.00	29.00	5.00	12.00	12.00	17.24	41.38	58.62	29.41	-0.41
Temperature	Predator	640.00	1000.00	20.00	2.00	29.00	4.00	10.00	15.00	13.79	34.48	48.28	28.57	-0.43
Temperature	Predator	640.00	1000.00	20.00	3.00	28.00	3.00	10.00	15.00	10.71	35.71	46.43	23.08	-0.54
Temperature	Predator	640.00	1000.00	25.00	1.00	25.00	6.00	10.00	9.00	24.00	40.00	64.00	37.50	-0.25
Temperature	Predator	640.00	1000.00	25.00	2.00	28.00	10.00	10.00	8.00	35.71	35.71	71.43	50.00	0.00
Temperature	Predator	640.00	1000.00	25.00	3.00	27.00	8.00	11.00	8.00	29.63	40.74	70.37	42.11	-0.16
Temperature	Predator	640.00	1000.00	30.00	1.00	29.00	10.00	10.00	9.00	34.48	34.48	68.97	50.00	0.00
Temperature	Predator	640.00	1000.00	30.00	2.00	25.00	10.00	6.00	9.00	40.00	24.00	64.00	62.50	0.25
Temperature	Predator	640.00	1000.00	30.00	3.00	26.00	10.00	8.00	8.00	38.46	30.77	69.23	55.56	0.11
Temperature	Predator	640.00	1000.00	35.00	1.00	23.00	8.00	3.00	12.00	34.78	13.04	47.83	72.73	0.45
Temperature	Predator	640.00	1000.00	35.00	2.00	25.00	9.00	4.00	12.00	36.00	16.00	52.00	69.23	0.38
Temperature	Predator	640.00	1000.00	35.00	3.00	24.00	8.00	4.00	12.00	33.33	16.67	50.00	66.67	0.33
Temperature	Whitefly	400.00	600.00	20.00	1.00	81.00	63.00	1.00	17.00	77.78	1.23	79.01	98.44	0.97
Temperature	Whitefly	400.00	600.00	20.00	2.00	94.00	80.00	0.00	14.00	85.11	0.00	85.11	100.00	1.00
Temperature	Whitefly	400.00	600.00	20.00	3.00	86.00	69.00	2.00	15.00	80.23	2.33	82.56	97.18	0.94
Temperature	Whitefly	400.00	600.00	25.00	1.00	47.00	43.00	1.00	3.00	91.49	2.13	93.62	97.73	0.95
Temperature	Whitefly	400.00	600.00	25.00	2.00	56.00	52.00	2.00	2.00	92.86	3.57	96.43	96.30	0.93

Temperature	Whitefly	400.00	600.00	25.00	3.00	55.00	50.00	1.00	4.00	90.91	1.82	92.73	98.04	0.96
Temperature	Whitefly	400.00	600.00	30.00	1.00	37.00	21.00	1.00	15.00	56.76	2.70	59.46	95.45	0.91
Temperature	Whitefly	400.00	600.00	30.00	2.00	55.00	36.00	2.00	17.00	65.45	3.64	69.09	94.74	0.89
Temperature	Whitefly	400.00	600.00	30.00	3.00	52.00	32.00	2.00	18.00	61.54	3.85	65.38	94.12	0.88
Temperature	Whitefly	400.00	600.00	35.00	1.00	70.00	66.00	2.00	2.00	94.29	2.86	97.14	97.06	0.94
Temperature	Whitefly	400.00	600.00	35.00	2.00	81.00	75.00	3.00	3.00	92.59	3.70	96.30	96.15	0.92
Temperature	Whitefly	400.00	600.00	35.00	3.00	70.00	65.00	2.00	3.00	92.86	2.86	95.71	97.01	0.94
Temperature	Whitefly	440.00	600.00	20.00	1.00	76.00	55.00	1.00	20.00	72.37	1.32	73.68	98.21	0.96
Temperature	Whitefly	440.00	600.00	20.00	2.00	77.00	60.00	2.00	15.00	77.92	2.60	80.52	96.77	0.94
Temperature	Whitefly	440.00	600.00	20.00	3.00	78.00	67.00	1.00	10.00	85.90	1.28	87.18	98.53	0.97
Temperature	Whitefly	440.00	600.00	25.00	1.00	56.00	50.00	1.00	5.00	89.29	1.79	91.07	98.04	0.96
Temperature	Whitefly	440.00	600.00	25.00	2.00	61.00	55.00	2.00	4.00	90.16	3.28	93.44	96.49	0.93
Temperature	Whitefly	440.00	600.00	25.00	3.00	62.00	58.00	1.00	3.00	93.55	1.61	95.16	98.31	0.97
Temperature	Whitefly	440.00	600.00	30.00	1.00	64.00	50.00	2.00	12.00	78.12	3.12	81.25	96.15	0.92
Temperature	Whitefly	440.00	600.00	30.00	2.00	67.00	57.00	3.00	7.00	85.07	4.48	89.55	95.00	0.90
Temperature	Whitefly	440.00	600.00	30.00	3.00	66.00	55.00	3.00	8.00	83.33	4.55	87.88	94.83	0.90
Temperature	Whitefly	440.00	600.00	35.00	1.00	59.00	50.00	1.00	8.00	84.75	1.69	86.44	98.04	0.96
Temperature	Whitefly	440.00	600.00	35.00	2.00	79.00	69.00	0.00	10.00	87.34	0.00	87.34	100.00	1.00
Temperature	Whitefly	440.00	600.00	35.00	3.00	75.00	65.00	2.00	8.00	86.67	2.67	89.33	97.01	0.94
Temperature	Whitefly	480.00	400.00	20.00	1.00	67.00	59.00	1.00	7.00	88.06	1.49	89.55	98.33	0.97
Temperature	Whitefly	480.00	400.00	20.00	2.00	77.00	65.00	2.00	10.00	84.42	2.60	87.01	97.01	0.94

Temperature	Whitefly	480.00	400.00	20.00	3.00	62.00	55.00	1.00	6.00	88.71	1.61	90.32	98.21	0.96
Temperature	Whitefly	480.00	400.00	25.00	1.00	93.00	67.00	6.00	20.00	72.04	6.45	78.49	91.78	0.84
Temperature	Whitefly	480.00	400.00	25.00	2.00	44.00	35.00	1.00	8.00	79.55	2.27	81.82	97.22	0.94
Temperature	Whitefly	480.00	400.00	25.00	3.00	83.00	65.00	3.00	15.00	78.31	3.61	81.93	95.59	0.91
Temperature	Whitefly	480.00	400.00	30.00	1.00	70.00	56.00	3.00	11.00	80.00	4.29	84.29	94.92	0.90
Temperature	Whitefly	480.00	400.00	30.00	2.00	86.00	71.00	5.00	10.00	82.56	5.81	88.37	93.42	0.87
Temperature	Whitefly	480.00	400.00	30.00	3.00	79.00	65.00	2.00	12.00	82.28	2.53	84.81	97.01	0.94
Temperature	Whitefly	480.00	400.00	35.00	1.00	75.00	54.00	15.00	6.00	72.00	20.00	92.00	78.26	0.57
Temperature	Whitefly	480.00	400.00	35.00	2.00	68.00	50.00	10.00	8.00	73.53	14.71	88.24	83.33	0.67
Temperature	Whitefly	480.00	400.00	35.00	3.00	70.00	52.00	10.00	8.00	74.29	14.29	88.57	83.87	0.68
Temperature	Whitefly	480.00	800.00	20.00	1.00	72.00	62.00	0.00	10.00	86.11	0.00	86.11	100.00	1.00
Temperature	Whitefly	480.00	800.00	20.00	2.00	69.00	60.00	3.00	6.00	86.96	4.35	91.30	95.24	0.90
Temperature	Whitefly	480.00	800.00	20.00	3.00	78.00	70.00	2.00	6.00	89.74	2.56	92.31	97.22	0.94
Temperature	Whitefly	480.00	800.00	25.00	1.00	78.00	65.00	3.00	10.00	83.33	3.85	87.18	95.59	0.91
Temperature	Whitefly	480.00	800.00	25.00	2.00	81.00	69.00	2.00	10.00	85.19	2.47	87.65	97.18	0.94
Temperature	Whitefly	480.00	800.00	25.00	3.00	80.00	65.00	3.00	12.00	81.25	3.75	85.00	95.59	0.91
Temperature	Whitefly	480.00	800.00	30.00	1.00	73.00	66.00	1.00	6.00	90.41	1.37	91.78	98.51	0.97
Temperature	Whitefly	480.00	800.00	30.00	2.00	75.00	65.00	2.00	8.00	86.67	2.67	89.33	97.01	0.94
Temperature	Whitefly	480.00	800.00	30.00	3.00	68.00	60.00	1.00	7.00	88.24	1.47	89.71	98.36	0.97
Temperature	Whitefly	480.00	800.00	35.00	1.00	65.00	55.00	8.00	2.00	84.62	12.31	96.92	87.30	0.75
Temperature	Whitefly	480.00	800.00	35.00	2.00	78.00	68.00	5.00	5.00	87.18	6.41	93.59	93.15	0.86

Temperature	Whitefly	480.00	800.00	35.00	3.00	68.00	60.00	5.00	3.00	88.24	7.35	95.59	92.31	0.85
Temperature	Whitefly	520.00	400.00	20.00	1.00	77.00	50.00	2.00	25.00	64.94	2.60	67.53	96.15	0.92
Temperature	Whitefly	520.00	400.00	20.00	2.00	78.00	46.00	2.00	30.00	58.97	2.56	61.54	95.83	0.92
Temperature	Whitefly	520.00	400.00	20.00	3.00	72.00	50.00	2.00	20.00	69.44	2.78	72.22	96.15	0.92
Temperature	Whitefly	520.00	400.00	25.00	1.00	68.00	36.00	1.00	31.00	52.94	1.47	54.41	97.30	0.95
Temperature	Whitefly	520.00	400.00	25.00	2.00	83.00	38.00	0.00	45.00	45.78	0.00	45.78	100.00	1.00
Temperature	Whitefly	520.00	400.00	25.00	3.00	78.00	35.00	1.00	42.00	44.87	1.28	46.15	97.22	0.94
Temperature	Whitefly	520.00	400.00	30.00	1.00	74.00	63.00	3.00	8.00	85.14	4.05	89.19	95.45	0.91
Temperature	Whitefly	520.00	400.00	30.00	2.00	73.00	65.00	4.00	4.00	89.04	5.48	94.52	94.20	0.88
Temperature	Whitefly	520.00	400.00	30.00	3.00	68.00	60.00	4.00	4.00	88.24	5.88	94.12	93.75	0.88
Temperature	Whitefly	520.00	400.00	35.00	1.00	59.00	50.00	6.00	3.00	84.75	10.17	94.92	89.29	0.79
Temperature	Whitefly	520.00	400.00	35.00	2.00	57.00	46.00	10.00	1.00	80.70	17.54	98.25	82.14	0.64
Temperature	Whitefly	520.00	400.00	35.00	3.00	57.00	48.00	7.00	2.00	84.21	12.28	96.49	87.27	0.75
Temperature	Whitefly	520.00	800.00	20.00	1.00	92.00	75.00	4.00	13.00	81.52	4.35	85.87	94.94	0.90
Temperature	Whitefly	520.00	800.00	20.00	2.00	70.00	65.00	1.00	4.00	92.86	1.43	94.29	98.48	0.97
Temperature	Whitefly	520.00	800.00	20.00	3.00	95.00	77.00	2.00	16.00	81.05	2.11	83.16	97.47	0.95
Temperature	Whitefly	520.00	800.00	25.00	1.00	74.00	56.00	2.00	16.00	75.68	2.70	78.38	96.55	0.93
Temperature	Whitefly	520.00	800.00	25.00	2.00	79.00	44.00	0.00	35.00	55.70	0.00	55.70	100.00	1.00
Temperature	Whitefly	520.00	800.00	25.00	3.00	82.00	50.00	2.00	30.00	60.98	2.44	63.41	96.15	0.92
Temperature	Whitefly	520.00	800.00	30.00	1.00	75.00	66.00	5.00	4.00	88.00	6.67	94.67	92.96	0.86
Temperature	Whitefly	520.00	800.00	30.00	2.00	66.00	60.00	3.00	3.00	90.91	4.55	95.45	95.24	0.90

Temperature	Whitefly	520.00	800.00	30.00	3.00	68.00	62.00	2.00	4.00	91.18	2.94	94.12	96.88	0.94
Temperature	Whitefly	520.00	800.00	35.00	1.00	61.00	52.00	8.00	1.00	85.25	13.11	98.36	86.67	0.73
Temperature	Whitefly	520.00	800.00	35.00	2.00	66.00	60.00	5.00	1.00	90.91	7.58	98.48	92.31	0.85
Temperature	Whitefly	520.00	800.00	35.00	3.00	63.00	55.00	7.00	1.00	87.30	11.11	98.41	88.71	0.77
Temperature	Whitefly	600.00	1000.00	20.00	1.00	92.00	42.00	2.00	48.00	45.65	2.17	47.83	95.45	0.91
Temperature	Whitefly	600.00	1000.00	20.00	2.00	94.00	42.00	2.00	50.00	44.68	2.13	46.81	95.45	0.91
Temperature	Whitefly	600.00	1000.00	20.00	3.00	91.00	40.00	4.00	47.00	43.96	4.40	48.35	90.91	0.82
Temperature	Whitefly	600.00	1000.00	25.00	1.00	59.00	37.00	2.00	20.00	62.71	3.39	66.10	94.87	0.90
Temperature	Whitefly	600.00	1000.00	25.00	2.00	54.00	30.00	2.00	22.00	55.56	3.70	59.26	93.75	0.88
Temperature	Whitefly	600.00	1000.00	25.00	3.00	61.00	35.00	1.00	25.00	57.38	1.64	59.02	97.22	0.94
Temperature	Whitefly	600.00	1000.00	30.00	1.00	60.00	40.00	10.00	10.00	66.67	16.67	83.33	80.00	0.60
Temperature	Whitefly	600.00	1000.00	30.00	2.00	66.00	36.00	15.00	15.00	54.55	22.73	77.27	70.59	0.41
Temperature	Whitefly	600.00	1000.00	30.00	3.00	61.00	39.00	12.00	10.00	63.93	19.67	83.61	76.47	0.53
Temperature	Whitefly	600.00	1000.00	35.00	1.00	73.00	46.00	12.00	15.00	63.01	16.44	79.45	79.31	0.59
Temperature	Whitefly	600.00	1000.00	35.00	2.00	85.00	60.00	15.00	10.00	70.59	17.65	88.24	80.00	0.60
Temperature	Whitefly	600.00	1000.00	35.00	3.00	77.00	55.00	10.00	12.00	71.43	12.99	84.42	84.62	0.69
Temperature	Whitefly	640.00	1000.00	20.00	1.00	80.00	37.00	3.00	40.00	46.25	3.75	50.00	92.50	0.85
Temperature	Whitefly	640.00	1000.00	20.00	2.00	78.00	35.00	5.00	38.00	44.87	6.41	51.28	87.50	0.75
Temperature	Whitefly	640.00	1000.00	20.00	3.00	86.00	37.00	4.00	45.00	43.02	4.65	47.67	90.24	0.80
Temperature	Whitefly	640.00	1000.00	25.00	1.00	55.00	50.00	1.00	4.00	90.91	1.82	92.73	98.04	0.96
Temperature	Whitefly	640.00	1000.00	25.00	2.00	67.00	62.00	2.00	3.00	92.54	2.99	95.52	96.88	0.94

Temperature	Whitefly	640.00	1000.00	25.00	3.00	57.00	55.00	0.00	2.00	96.49	0.00	96.49	100.00	1.00
Temperature	Whitefly	640.00	1000.00	30.00	1.00	65.00	36.00	18.00	11.00	55.38	27.69	83.08	66.67	0.33
Temperature	Whitefly	640.00	1000.00	30.00	2.00	68.00	42.00	16.00	10.00	61.76	23.53	85.29	72.41	0.45
Temperature	Whitefly	640.00	1000.00	30.00	3.00	64.00	40.00	15.00	9.00	62.50	23.44	85.94	72.73	0.45
Temperature	Whitefly	640.00	1000.00	35.00	1.00	73.00	40.00	17.00	16.00	54.79	23.29	78.08	70.18	0.40
Temperature	Whitefly	640.00	1000.00	35.00	2.00	61.00	31.00	10.00	20.00	50.82	16.39	67.21	75.61	0.51
Temperature	Whitefly	640.00	1000.00	35.00	3.00	64.00	34.00	12.00	18.00	53.12	18.75	71.88	73.91	0.48

Table S3. Omnibus Wald chi-square tests from the raw-count binomial GLM reanalysis.

Assay	Species	Endpoint	Term	Chi2	P Value	DF Constraint
Screening	Whitefly	Positive Phototaxis	Intercept	84.22	4.42E-20	1.00
Screening	Whitefly	Positive Phototaxis	C(Wavelength (nm))	121.71	7.12E-24	6.00
Screening	Whitefly	Positive Phototaxis	C(Irradiance (μW cm−2))	193.71	6.28E-40	5.00
Screening	Whitefly	Positive Phototaxis	C(Wavelength (nm)):C(Irradiance (μW cm−2))	655.22	1.03E-118	30.00
Screening	Whitefly	Negative Phototaxis	Intercept	5.49E-07	1.00	1.00
Screening	Whitefly	Negative Phototaxis	C(Wavelength (nm))	15.62	0.02	6.00
Screening	Whitefly	Negative Phototaxis	C(Irradiance (μW cm−2))	1.97	0.85	5.00
Screening	Whitefly	Negative Phototaxis	C(Wavelength (nm)):C(Irradiance (μW cm−2))	39.24	0.12	30.00
Temperature	Whitefly	Positive Phototaxis	Intercept	85.39	2.44E-20	1.00
Temperature	Whitefly	Positive Phototaxis	C(Temperature (°C))	61.87	2.34E-13	3.00
Temperature	Whitefly	Positive Phototaxis	C(setting)	248.22	6.64E-50	7.00
Temperature	Whitefly	Positive Phototaxis	C(Temperature (°C)):C(setting)	311.79	1.26E-53	21.00
Temperature	Whitefly	Negative Phototaxis	Intercept	58.84	1.71E-14	1.00
Temperature	Whitefly	Negative Phototaxis	C(Temperature (°C))	2.76	0.43	3.00
Temperature	Whitefly	Negative Phototaxis	C(setting)	8.09	0.32	7.00
Temperature	Whitefly	Negative Phototaxis	C(Temperature (°C)):C(setting)	62.63	5.06E-06	21.00
Temperature	Whitefly	Response Rate	Intercept	90.10	2.26E-21	1.00
Temperature	Whitefly	Response Rate	C(Temperature (°C))	63.63	9.85E-14	3.00
Temperature	Whitefly	Response Rate	C(setting)	237.12	1.53E-47	7.00

Assay	Species	Endpoint	Term	Chi2	P Value	DF Constraint
Temperature	Whitefly	Response Rate	C(Temperature (°C)):C(setting)	346.88	8.28E-61	21.00
Screening	Predator	Positive Phototaxis	Intercept	25.07	5.54E-07	1.00
Screening	Predator	Positive Phototaxis	C(Wavelength (nm))	24.59	0.00	6.00
Screening	Predator	Positive Phototaxis	C(Irradiance (μW cm−2))	13.85	0.02	5.00
Screening	Predator	Positive Phototaxis	C(Wavelength (nm)):C(Irradiance (μW cm−2))	62.92	0.00	30.00
Screening	Predator	Negative Phototaxis	Intercept	0.45	0.50	1.00
Screening	Predator	Negative Phototaxis	C(Wavelength (nm))	34.18	6.21E-06	6.00
Screening	Predator	Negative Phototaxis	C(Irradiance (μW cm−2))	6.26	0.28	5.00
Screening	Predator	Negative Phototaxis	C(Wavelength (nm)):C(Irradiance (μW cm−2))	49.48	0.01	30.00
Temperature	Predator	Positive Phototaxis	Intercept	31.42	2.07E-08	1.00
Temperature	Predator	Positive Phototaxis	C(Temperature (°C))	16.76	0.00	3.00
Temperature	Predator	Positive Phototaxis	C(setting)	34.23	1.56E-05	7.00
Temperature	Predator	Positive Phototaxis	C(Temperature (°C)):C(setting)	68.28	6.62E-07	21.00
Temperature	Predator	Negative Phototaxis	Intercept	36.68	1.39E-09	1.00
Temperature	Predator	Negative Phototaxis	C(Temperature (°C))	66.18	2.80E-14	3.00
Temperature	Predator	Negative Phototaxis	C(setting)	75.26	1.27E-13	7.00
Temperature	Predator	Negative Phototaxis	C(Temperature (°C)):C(setting)	84.92	1.20E-09	21.00
Temperature	Predator	Response Rate	Intercept	35.82	2.16E-09	1.00
Temperature	Predator	Response Rate	C(Temperature (°C))	45.42	7.52E-10	3.00
Temperature	Predator	Response Rate	C(setting)	44.53	1.69E-07	7.00

Assay	Species	Endpoint	Term	Chi2	P Value	DF Constraint
Temperature	Predator	Response Rate	C(Temperature (°C)):C(setting)	76.50	3.08E-08	21.00

Table S4. Temperature-specific whitefly summary table used for ranking and decision analysis.

Species	Temperature (°C)	Wavelength (nm)	Irradiance ($\mu W\ cm^{-2}$)	Replicates id	Positive mean (%)	Positive se (%)	Negative mean (%)	Negative se (%)	Response rate mean (%)	Response rate se (%)	Light preference mean (%)	Light preference se (%)	Net attraction (percentage points)	Negative > positive	Rank by positive	Rank by net attraction
Whitefly	20.00	400.00	600.00	3.00	81.04	2.15	1.19	0.67	82.23	1.77	98.54	0.81	79.85	False	4.00	4.00
Whitefly	20.00	440.00	600.00	3.00	78.73	3.93	1.73	0.43	80.46	3.90	97.84	0.54	77.00	False	5.00	5.00
Whitefly	20.00	480.00	400.00	3.00	87.06	1.34	1.90	0.35	88.96	1.00	97.85	0.42	85.16	False	2.00	2.00
Whitefly	20.00	480.00	800.00	3.00	87.60	1.10	2.30	1.26	89.91	1.92	97.49	1.38	85.30	False	1.00	1.00
Whitefly	20.00	520.00	400.00	3.00	64.45	3.03	2.65	0.07	67.10	3.09	96.05	0.11	61.80	False	6.00	6.00
Whitefly	20.00	520.00	800.00	3.00	85.14	3.86	2.63	0.88	87.77	3.35	96.96	1.05	82.52	False	3.00	3.00
Whitefly	20.00	600.00	1000.00	3.00	44.76	0.49	2.90	0.75	47.66	0.45	93.94	1.52	41.86	False	7.00	7.00
Whitefly	20.00	640.00	1000.00	3.00	44.72	0.93	4.94	0.78	49.65	1.06	90.08	1.45	39.78	False	8.00	8.00
Whitefly	25.00	400.00	600.00	3.00	91.75	0.58	2.51	0.54	94.26	1.12	97.35	0.54	89.25	False	2.00	2.00
Whitefly	25.00	440.00	600.00	3.00	91.00	1.30	2.23	0.53	93.23	1.19	97.61	0.57	88.77	False	3.00	3.00
Whitefly	25.00	480.00	400.00	3.00	76.63	2.32	4.11	1.23	80.75	1.13	94.86	1.61	72.52	False	5.00	5.00
Whitefly	25.00	480.00	800.00	3.00	83.26	1.14	3.36	0.44	86.61	0.82	96.12	0.53	79.90	False	4.00	4.00
Whitefly	25.00	520.00	400.00	3.00	47.87	2.55	0.92	0.46	48.78	2.82	98.17	0.91	46.95	False	8.00	8.00
Whitefly	25.00	520.00	800.00	3.00	64.12	5.98	1.71	0.86	65.83	6.66	97.57	1.22	62.40	False	6.00	6.00
Whitefly	25.00	600.00	1000.00	3.00	58.55	2.15	2.91	0.64	61.46	2.32	95.28	1.02	55.64	False	7.00	7.00
Whitefly	25.00	640.00	1000.00	3.00	93.31	1.66	1.60	0.87	94.91	1.13	98.30	0.91	91.71	False	1.00	1.00

Species	Temperature (°C)	Wavelength (nm)	Irradiance (μW cm^{-2})	Replicates id	Positive mean (%)	Positive se (%)	Negative mean (%)	Negative se (%)	Response rate mean (%)	Response rate se (%)	Light preference mean (%)	Light preference se (%)	Net attraction (percentage points)	Negative > positive	Rank by positive	Rank by net attraction
Whitefly	30.00	400.00	600.00	3.00	61.25	2.52	3.40	0.35	64.64	2.80	94.77	0.39	57.85	False	7.00	6.00
Whitefly	30.00	440.00	600.00	3.00	82.18	2.09	4.05	0.46	86.23	2.54	95.33	0.42	78.13	False	4.00	4.00
Whitefly	30.00	480.00	400.00	3.00	81.61	0.81	4.21	0.95	85.82	1.28	95.12	1.04	77.40	False	5.00	5.00
Whitefly	30.00	480.00	800.00	3.00	88.44	1.09	1.84	0.42	90.27	0.76	97.96	0.47	86.60	False	2.00	1.00
Whitefly	30.00	520.00	400.00	3.00	87.47	1.19	5.14	0.55	92.61	1.71	94.47	0.51	82.33	False	3.00	3.00
Whitefly	30.00	520.00	800.00	3.00	90.03	1.02	4.72	1.08	94.75	0.39	95.02	1.14	85.31	False	1.00	2.00
Whitefly	30.00	600.00	1000.00	3.00	61.72	3.67	19.69	1.75	81.40	2.07	75.69	2.75	42.03	False	6.00	7.00
Whitefly	30.00	640.00	1000.00	3.00	59.88	2.26	24.89	1.40	84.77	0.87	70.60	1.97	35.00	False	8.00	8.00
Whitefly	35.00	400.00	600.00	3.00	93.25	0.53	3.14	0.28	96.38	0.41	96.74	0.29	90.11	False	1.00	1.00
Whitefly	35.00	440.00	600.00	3.00	86.25	0.78	1.45	0.78	87.71	0.85	98.35	0.88	84.80	False	4.00	2.00
Whitefly	35.00	480.00	400.00	3.00	73.27	0.67	16.33	1.84	89.60	1.20	81.82	1.79	56.94	False	6.00	6.00
Whitefly	35.00	480.00	800.00	3.00	86.68	1.07	8.69	1.83	95.37	0.97	90.92	1.83	77.99	False	3.00	3.00
Whitefly	35.00	520.00	400.00	3.00	83.22	1.27	13.33	2.19	96.55	0.96	86.23	2.13	69.89	False	5.00	5.00
Whitefly	35.00	520.00	800.00	3.00	87.82	1.66	10.60	1.62	98.42	0.04	89.23	1.65	77.22	False	2.00	4.00
Whitefly	35.00	600.00	1000.00	3.00	68.34	2.68	15.69	1.40	84.03	2.54	81.31	1.67	52.65	False	7.00	7.00
Whitefly	35.00	640.00	1000.00	3.00	52.91	1.15	19.48	2.02	72.39	3.15	73.23	1.61	33.44	False	8.00	8.00

Table S5. Temperature-specific predator summary table used for ranking and decision analysis.

Species	Temperature (°C)	Wavelength (nm)	Irradiance (μW cm^{-2})	Replicates id	Positive mean (%)	Positive se (%)	Negative mean (%)	Negative se (%)	Response rate mean (%)	Response rate se (%)	Light preference mean (%)	Light preference se (%)	Net attraction (percentage points)	Negative > positive	Rank by negative	Rank by net avoidance	Net avoidance (percentage points)
Predator	20.00	400.00	600.00	3.00	3.56	1.94	90.63	3.88	94.18	2.10	3.86	2.14	-87.07	True	1.00	1.00	87.07
Predator	20.00	440.00	600.00	3.00	1.45	1.45	65.24	2.14	66.69	2.58	2.08	2.08	-63.79	True	2.00	2.00	63.79
Predator	20.00	480.00	400.00	3.00	23.91	1.45	55.51	2.93	79.42	2.01	30.19	2.30	-31.59	True	3.00	3.00	31.59
Predator	20.00	480.00	800.00	3.00	19.70	5.67	51.15	0.57	70.85	5.96	26.93	5.30	-31.45	True	4.00	4.00	31.45
Predator	20.00	520.00	400.00	3.00	32.41	3.13	23.38	4.45	55.79	2.92	58.42	6.60	9.03	False	8.00	8.00	-9.03
Predator	20.00	520.00	800.00	3.00	16.98	1.66	38.73	2.82	55.71	2.97	30.56	2.78	-21.75	True	6.00	6.00	21.75
Predator	20.00	600.00	1000.00	3.00	30.51	0.26	41.54	2.35	72.05	2.45	42.43	1.30	-11.03	True	5.00	7.00	11.03
Predator	20.00	640.00	1000.00	3.00	13.92	1.89	37.19	2.12	51.11	3.79	27.02	1.99	-23.28	True	7.00	5.00	23.28
Predator	25.00	400.00	600.00	3.00	25.51	4.38	55.82	4.94	81.32	0.67	31.44	5.64	-30.31	True	1.00	1.00	30.31
Predator	25.00	440.00	600.00	3.00	36.18	2.91	47.58	3.13	83.76	1.15	43.21	3.49	-11.40	True	2.00	2.00	11.40
Predator	25.00	480.00	400.00	3.00	41.58	3.08	24.45	1.61	66.03	3.67	62.88	2.27	17.12	False	6.00	6.00	-17.12
Predator	25.00	480.00	800.00	3.00	28.57	4.12	33.33	2.38	61.90	6.30	45.77	2.17	-4.76	True	5.00	4.00	4.76
Predator	25.00	520.00	400.00	3.00	45.26	1.41	14.84	2.53	60.10	3.40	75.61	3.09	30.42	False	8.00	8.00	-30.42
Predator	25.00	520.00	800.00	3.00	48.04	2.75	23.27	3.30	71.31	1.03	67.44	4.39	24.77	False	7.00	7.00	-24.77
Predator	25.00	600.00	1000.00	3.00	36.24	4.59	39.73	1.69	75.97	2.94	47.39	4.20	-3.48	True	3.00	5.00	3.48
Predator	25.00	640.00	1000.00	3.00	29.78	3.38	38.82	1.57	68.60	2.32	43.20	3.65	-9.04	True	4.00	3.00	9.04

Species	Temperature (°C)	Wavelength (nm)	Irradiance (μW cm^{-2})	Replicates id	Positive mean (%)	Positive se (%)	Negative mean (%)	Negative se (%)	Response rate mean (%)	Response rate se (%)	Light preference mean (%)	Light preference se (%)	Net attraction (percentage points)	Negative > positive	Rank by negative	Rank by net avoidance	Net avoidance (percentage points)
Predator	30.00	400.00	600.00	3.00	32.92	0.78	42.28	3.64	75.21	4.30	43.96	1.74	-9.36	True	1.00	1.00	9.36
Predator	30.00	440.00	600.00	3.00	45.13	1.42	22.44	2.21	67.58	3.41	66.94	1.65	22.69	False	7.00	6.00	-22.69
Predator	30.00	480.00	400.00	3.00	43.68	2.82	33.71	1.82	77.40	4.61	56.41	0.46	9.97	False	3.00	3.00	-9.97
Predator	30.00	480.00	800.00	3.00	51.06	1.59	23.63	4.48	74.69	2.90	68.73	4.72	27.43	False	6.00	7.00	-27.43
Predator	30.00	520.00	400.00	3.00	55.02	2.53	12.38	1.20	67.39	1.34	81.55	2.10	42.64	False	8.00	8.00	-42.64
Predator	30.00	520.00	800.00	3.00	41.58	3.08	24.18	4.40	65.75	2.95	63.52	5.88	17.40	False	5.00	5.00	-17.40
Predator	30.00	600.00	1000.00	3.00	46.05	0.61	33.72	1.96	79.77	1.94	57.80	1.54	12.34	False	2.00	4.00	-12.34
Predator	30.00	640.00	1000.00	3.00	37.65	1.64	29.75	3.07	67.40	1.70	56.02	3.62	7.90	False	4.00	2.00	-7.90
Predator	35.00	400.00	600.00	3.00	26.13	1.97	19.02	0.86	45.15	2.27	57.74	2.04	7.10	False	6.00	4.00	-7.10
Predator	35.00	440.00	600.00	3.00	17.97	2.06	25.11	3.46	43.07	3.94	42.06	4.83	-7.14	True	3.00	2.00	7.14
Predator	35.00	480.00	400.00	3.00	40.60	3.83	24.89	3.20	65.49	0.69	61.89	5.21	15.71	False	4.00	5.00	-15.71
Predator	35.00	480.00	800.00	3.00	56.57	5.85	17.61	1.85	74.18	4.04	75.87	3.67	38.96	False	7.00	8.00	-38.96
Predator	35.00	520.00	400.00	3.00	21.03	2.53	30.26	3.47	51.28	1.73	41.21	5.61	-9.23	True	2.00	1.00	9.23
Predator	35.00	520.00	800.00	3.00	49.92	4.50	21.61	3.09	71.53	2.57	69.64	4.83	28.31	False	5.00	7.00	-28.31
Predator	35.00	600.00	1000.00	3.00	30.51	1.44	30.63	1.41	61.15	2.00	49.90	1.61	-0.12	True	1.00	3.00	0.12
Predator	35.00	640.00	1000.00	3.00	34.71	0.77	15.24	1.11	49.94	1.21	69.54	1.76	19.47	False	8.00	6.00	-19.47

Table S6. All candidate coordinated-setting scores before strict decision filtering.

Temperature (°C)	Wavelength (nm)	Irradiance (μW cm^{-2})	Whitefly positive mean (%)	Whitefly negative mean (%)	Whitefly net attraction (percentage points)	Whitefly rank by positive	Predator positive mean (%)	Predator negative mean (%)	Predator net avoidance (percentage points)	Predator rank by negative	Strict avoidance flag	Coordinated balance score
20.00	400.00	600.00	81.04	1.19	79.85	4.00	3.56	90.63	87.07	1.00	True	166.93
20.00	440.00	600.00	78.73	1.73	77.00	5.00	1.45	65.24	63.79	2.00	True	140.79
20.00	480.00	400.00	87.06	1.90	85.16	2.00	23.91	55.51	31.59	3.00	True	116.75
20.00	480.00	800.00	87.60	2.30	85.30	1.00	19.70	51.15	31.45	4.00	True	116.74
20.00	520.00	400.00	64.45	2.65	61.80	6.00	32.41	23.38	-9.03	8.00	False	52.78
20.00	520.00	800.00	85.14	2.63	82.52	3.00	16.98	38.73	21.75	6.00	True	104.26
20.00	600.00	1000.00	44.76	2.90	41.86	7.00	30.51	41.54	11.03	5.00	True	52.89
20.00	640.00	1000.00	44.72	4.94	39.78	8.00	13.92	37.19	23.28	7.00	True	63.05
25.00	400.00	600.00	91.75	2.51	89.25	2.00	25.51	55.82	30.31	1.00	True	119.56
25.00	440.00	600.00	91.00	2.23	88.77	3.00	36.18	47.58	11.40	2.00	True	100.17
25.00	480.00	400.00	76.63	4.11	72.52	5.00	41.58	24.45	-17.12	6.00	False	55.40
25.00	480.00	800.00	83.26	3.36	79.90	4.00	28.57	33.33	4.76	5.00	True	84.66
25.00	520.00	400.00	47.87	0.92	46.95	8.00	45.26	14.84	-30.42	8.00	False	16.53
25.00	520.00	800.00	64.12	1.71	62.40	6.00	48.04	23.27	-24.77	7.00	False	37.63
25.00	600.00	1000.00	58.55	2.91	55.64	7.00	36.24	39.73	3.48	3.00	True	59.12
25.00	640.00	1000.00	93.31	1.60	91.71	1.00	29.78	38.82	9.04	4.00	True	100.75
30.00	400.00	600.00	61.25	3.40	57.85	7.00	32.92	42.28	9.36	1.00	True	67.21

30.00	440.00	600.00	82.18	4.05	78.13	4.00	45.13	22.44	-22.69	7.00	False	55.44
30.00	480.00	400.00	81.61	4.21	77.40	5.00	43.68	33.71	-9.97	3.00	False	67.43
30.00	480.00	800.00	88.44	1.84	86.60	2.00	51.06	23.63	-27.43	6.00	False	59.17
30.00	520.00	400.00	87.47	5.14	82.33	3.00	55.02	12.38	-42.64	8.00	False	39.69
30.00	520.00	800.00	90.03	4.72	85.31	1.00	41.58	24.18	-17.40	5.00	False	67.91
30.00	600.00	1000.00	61.72	19.69	42.03	6.00	46.05	33.72	-12.34	2.00	False	29.69
30.00	640.00	1000.00	59.88	24.89	35.00	8.00	37.65	29.75	-7.90	4.00	False	27.10
35.00	400.00	600.00	93.25	3.14	90.11	1.00	26.13	19.02	-7.10	6.00	False	83.00
35.00	440.00	600.00	86.25	1.45	84.80	4.00	17.97	25.11	7.14	3.00	True	91.94
35.00	480.00	400.00	73.27	16.33	56.94	6.00	40.60	24.89	-15.71	4.00	False	41.24
35.00	480.00	800.00	86.68	8.69	77.99	3.00	56.57	17.61	-38.96	7.00	False	39.03
35.00	520.00	400.00	83.22	13.33	69.89	5.00	21.03	30.26	9.23	2.00	True	79.12
35.00	520.00	800.00	87.82	10.60	77.22	2.00	49.92	21.61	-28.31	5.00	False	48.90
35.00	600.00	1000.00	68.34	15.69	52.65	7.00	30.51	30.63	0.12	1.00	True	52.78
35.00	640.00	1000.00	52.91	19.48	33.44	8.00	34.71	15.24	-19.47	8.00	False	13.97

Table S7. Definitions of supplementary behavioral metrics and detailed decision rules used for ranking candidate LED settings.

Item	Definition / Rule
Response rate (%)	Calculated as (light arm + dark arm) / total × 100, where total denotes the realized total counted per replicate. This metric was used to indicate the proportion of adults that entered either choice arm.
Light preference among responders (%)	Calculated as light arm / (light arm + dark arm) × 100. This metric describes directional preference among adults that left the base arm.
Choice index among responders	Calculated as (light arm - dark arm) / (light arm + dark arm), ranging from -1 to 1. Positive values indicate preference for the light arm, whereas negative values indicate preference for the dark arm.
Screening-stage whitefly ranking	For each irradiance level, the peak whitefly setting was defined as the wavelength with the highest mean positive phototaxis. If two settings were numerically similar, lower mean negative phototaxis was used as a tiebreaker.
Temperature-stage whitefly ranking	At each temperature, the top whitefly-attraction setting was defined as the treatment with the highest mean positive phototaxis.
Temperature-stage predator-safe ranking	Candidate predator-safe settings first had to satisfy a strict-avoidance criterion in which mean negative phototaxis exceeded mean positive phototaxis. Treatments meeting this condition were then ranked by predator net avoidance.
Predator net avoidance	Calculated as mean negative phototaxis - mean positive phototaxis. Larger values indicate stronger predator avoidance under the strict criterion.
Whitefly net attraction	Calculated as mean positive phototaxis - mean negative phototaxis. Larger values indicate stronger whitefly-oriented attraction.
Coordinated-use candidate	The strict coordinated-use candidate at each temperature was selected from the strict-avoidance treatments as the setting with the highest coordinated balance score.
Coordinated balance score	Calculated as whitefly net attraction + predator net avoidance. This score was used to jointly evaluate whitefly attraction and predator avoidance among strict-avoidance treatments.
Selection of LED settings for temperature-stage assays	The LED settings used in the temperature-stage assays were selected from the wavelength–irradiance screening stage. Candidate settings were chosen to represent treatments with high positive phototaxis in *B. tabaci*, strong negative phototaxis or low positive phototaxis in *S. japonicum*, or potential coordinated performance between whitefly attraction and predator avoidance.

Item	Definition / Rule
Use of ranking and points	Rankings and coordinated scores were used as application-oriented decision aids to compare candidate LED settings after the screening stage. They were used to balance pest attraction and predator avoidance, rather than as independent statistical tests.
Statistical interpretation of “best” settings	The terms “best”, “highest”, or “strongest” in the decision tables refer to treatment means ranked according to the predefined decision rules. Statistical effects of wavelength, irradiance, temperature, and their interactions were evaluated separately using binomial GLMs and Wald chi-square tests. Therefore, ranking-based choices should be interpreted together with the model results and not as evidence that every ranked treatment differed significantly from all others.
